# *Trypanosoma cruzi *mitochondrial maxicircles display species- and strain-specific variation and a conserved element in the non-coding region

**DOI:** 10.1186/1471-2164-7-60

**Published:** 2006-03-22

**Authors:** Scott J Westenberger, Gustavo C Cerqueira, Najib M El-Sayed, Bianca Zingales, David A Campbell, Nancy R Sturm

**Affiliations:** 1 Department of Microbiology, Immunology & Molecular Genetics, David Geffen School of Medicine, University of California at Los Angeles 90095, USA; 2 Department of Parasite Genomics, The Institute for Genomic Research, Rockville, MD 20850, USA; 3 Departamento de Bioquímica e Imunologia, Universidade Federal de Minas Gerais, Minas Gerais, Brazil; 4 Departamento de Bioquímica, Instituto de Química, Universidade de São Paulo, São Paulo, Brazil

## Abstract

**Background:**

The mitochondrial DNA of kinetoplastid flagellates is distinctive in the eukaryotic world due to its massive size, complex form and large sequence content. Comprised of catenated maxicircles that contain rRNA and protein-coding genes and thousands of heterogeneous minicircles encoding small guide RNAs, the kinetoplast network has evolved along with an extreme form of mRNA processing in the form of uridine insertion and deletion RNA editing. Many maxicircle-encoded mRNAs cannot be translated without this post-transcriptional sequence modification.

**Results:**

We present the complete sequence and annotation of the *Trypanosoma cruzi *maxicircles for the CL Brener and Esmeraldo strains. Gene order is syntenic with *Trypanosoma brucei *and *Leishmania tarentolae *maxicircles. The non-coding components have strain-specific repetitive regions and a variable region that is unique for each strain with the exception of a conserved sequence element that may serve as an origin of replication, but shows no sequence identity with *L. tarentolae *or *T. brucei*. Alternative assemblies of the variable region demonstrate intra-strain heterogeneity of the maxicircle population. The extent of mRNA editing required for particular genes approximates that seen in *T. brucei*. Extensively edited genes were more divergent among the genera than non-edited and rRNA genes. Esmeraldo contains a unique 236-bp deletion that removes the 5'-ends of *ND4 *and *CR4 *and the intergenic region. Esmeraldo shows additional insertions and deletions outside of areas edited in other species in *ND5*, *MURF1*, and *MURF2*, while CL Brener has a distinct insertion in *MURF2*.

**Conclusion:**

The CL Brener and Esmeraldo maxicircles represent two of three previously defined maxicircle clades and promise utility as taxonomic markers. Restoration of the disrupted reading frames might be accomplished by strain-specific RNA editing. Elements in the non-coding region may be important for replication, transcription, and anchoring of the maxicircle within the kinetoplast network.

## Background

The mitochondrial DNA referred to as the kinetoplast (kDNA) is a spectacular structure that comprises approximately 20–25% of the total cellular DNA in *Trypanosoma cruzi*, a member of the flagellated protozoans of the Order Kinetoplastida [1]. The equally dramatic process of RNA editing is also found in this specialized subcellular compartment, and the two are intimately associated. The kDNA is comprised of two classes of circular DNA molecules that are catenated and compressed into a disc-like structure. Maxicircles are the functional equivalent of the mitochondrial DNA of other eukaryotes, containing genes for mitochondrial rRNAs and hydrophobic mitochondrial proteins mostly involved in the membrane-bound oxidative phosphorylation pathway [2]. At first glance the maxicircle genomes appear to lack several genes that are hallmarks of other mitochondrial genomes, while other genes are missing elements key to their translation such as initiation codons or contiguous ORFs. Post-transcriptional uridine insertion/deletion RNA editing resolves most of these problematic issues, by creating start codons [3,4], correcting internal frameshifts (e.g., four uridines inserted in COII [5-7]), and extensively modifying otherwise unrecognizable mRNA transcripts to create entire ORFs [8] (e.g., 547 uridines inserted and 41 deleted in COIII of *Trypanosoma brucei *[9]). The mechanistic process of RNA editing is the subject of intense study in *T. brucei *and *Leishmania tarentolae *[10,11].

The heterogeneous minicircle population makes up the bulk of the kDNA mass with tens of thousands of copies per network, and carries the specific information for RNA editing in the form of guide RNAs (gRNAs). A handful of gRNAs are also encoded on the maxicircle, with 15 thus far identified in *L. tarentolae *and three in *T. brucei *[12]. The gRNAs interact with the mRNA templates through hybridization to dictate the precise location and number of uridine insertions or deletions; the interaction is tolerant of wobble base pairing of G-U, in addition to the standard Watson-Crick pairs. The level of sequence variability permissible in the gRNA pool with no loss of functional information is impressive, as any pairing with G or U residues in the mRNA are unaffected by transition mutations in the gRNA. This leads to tolerance of sequence divergence while maintaining function, as evidenced by the lack of cross-recognition of the gRNAs from two strains (SylvioX10 and CANIII) of *T. cruzi *[13]. The minicircle variable regions that encode the gRNAs have been used as molecular markers for strain genotyping and comparison [14]. The minicircle population of *T. cruzi *is highly variable between strains and evolves rapidly over time, allowing differentiation of closely related strains [15-18].

*T. cruzi *is the causative agent of human Chagas disease, affecting millions throughout the American continents, and is estimated to kill 100,000 people per year [19]. The infection is spread by introduction of vector insect contaminated feces into an open wound or mucus membrane of the victim. Once inside the human host, *T. cruzi *infects macrophages and replicates aggressively, producing high titers of parasite in the bloodstream that then spread throughout the tissues of the body. Approximately 5% of infected individuals die in this acute stage. Ultimately the infection will enter a quiescent phase that can last for decades, characterized by a low blood titer of the parasite and no overt symptoms. In 30% of the cases, clinical pathologies will eventually develop after 20+ years, reflecting a preference for cardiac and smooth muscle tissue by the parasite. Chronic phase chagasic patients may die from one of a suite of mega syndromes affecting the heart, esophagus, or colon. While the factors determining development of these specific syndromes is not understood, the genetics of both the host and the parasite play roles in the outcome [20].

Unraveling the genetics of *T. cruzi *has been a major endeavor for decades. Currently *T. cruzi *is partitioned into two groups [21] that can be subdivided into six subgroups or discrete typing units (DTUs) designated I, IIa, IIb, IIc, IId, and IIe [22-24]. Nuclear markers indicate that homozygous DTUs IIa and IIc are the result of a relatively ancient hybridization event between strains of DTUs I and IIb; the extensively heterozygous DTUs IId and IIe are products of a more recent hybridization event and possess two alleles similar to those found in DTUs IIb and IIc at most loci [25]. The reference strain chosen for the *T. cruzi *genome-sequencing project, CL Brener, is a member of DTU IIe. Sequencing of Esmeraldo, a homozygous DTU IIb strain, was undertaken to aid in resolution of ambiguity in the assembly of the CL Brener genome.

The influence of a persistent *T. cruzi *infection in development of chronic stage of Chagas disease is the subject of debate, since the direct cause of pathogenesis has yet to be determined conclusively [26-31]. Essentially, there are two alternative hypotheses: 1) the presence of the parasite itself results in destruction of infected tissues, versus 2) infection by the parasite triggers an autoimmune response against cells not necessarily harboring active infections. In addition to a multitude of protein antigens [32], an alternative stimulus for initiation of the autoimmune response has been proposed. During the cellular invasion process, DNA from the parasite is integrated into the host genome. The DNA species implicated in this horizontal transfer event is the minicircle [33,34]. Whether this is a benign byproduct of infection, or a potential link to Chagas disease pathology, the function of *T. cruzi *kDNA is relevant to understanding parasite biology and the host-parasite relationship.

Genetic variation in the *T. cruzi *maxicircle will be subject to more stringent selection pressure due to the presence of structural genes compared to the transition-tolerant gRNAs largely carried in the minicircles, and should provide phylogenetically meaningful markers. Maxicircle genes from *T. brucei *and *L. tarentolae *have been determined, showing that gene order, or synteny, is conserved [2]. Estimates of the total size of the *T. cruzi *maxicircle fall between 21 and 39 kb by various methods [35,36], and a fragment of maxicircle from the Tulahuen strain was sequenced [37]. Assorted fragments of maxicircles from several *T. cruzi *strains have been examined for taxonomic studies [38-41]. An extensive *T. cruzi *maxicircle survey examined a 1.25-kb fragment, identifying a correlation between the DTUs and three maxicircle clades [40]: clade A corresponded to DTU I, clade B to strains of DTUs IIa, IIc, IId, and IIe, and clade C was exclusive to DTU IIb strains. The same association was observed in similar analyses [38].

We present annotated sequences of the *T. cruzi *maxicircles for the CL Brener (DTU IIe) and Esmeraldo (DTU IIb) strains assembled from data generated by the TIGR-SBRI-KI *T. cruzi *Sequencing Consortium (TSK-TSC). The anticipated cohort of genes was present, and the non-coding regions of both strains were assembled. The coding region of each genome contained little or no single nucleotide polymorphism (SNP) variability, but do possess strain-specific insertion/deletion mutations (indels). Esmeraldo has a large deletion removing substantial portions of two adjacent genes. This study provides the framework for continued study of kDNA biology, RNA editing, and Chagas disease pathology in *T. cruzi*.

## Results

### Two *T. cruzi *maxicircle assemblies

The *T. cruzi *genome project took the approach of whole-genome shotgun sequencing of clones from libraries of size-selected fragments from a total cell DNA preparation with a minimum insert size of 5 kb. Clones were sequenced from both ends, providing between 500 bp and 1 kb of primary sequence and a physical link between opposing end-sequenced mate pairs from each clone [42]. A BLAST search of the primary reads from this project identified sequences of the CL Brener and Esmeraldo strains with identity to maxicircle coding regions from *T. cruzi *and *T. brucei*. The chromosome shotgun or large insert clone-based sequencing strategies employed by the *L. major *and *T. brucei *genome projects would preclude the capture of maxicircles.

**Figure 1:**
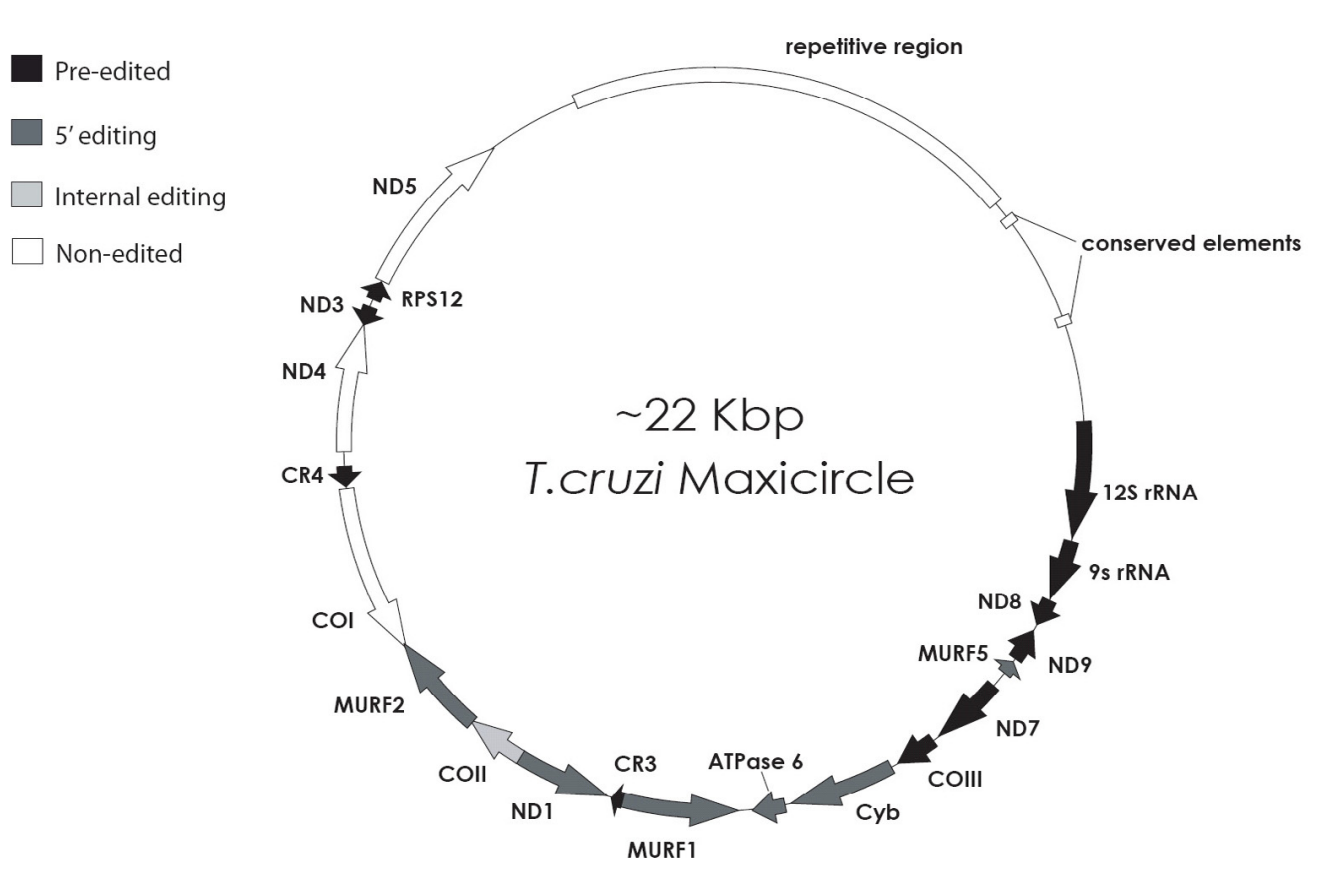
**The *T. cruzi *maxicircle of CL Brener and Esmeraldo. **All annotated genes are shown as arrows indicating coding direction. The non-coding regions of both genomes are distinct from one another, with the exception of a duplicated conserved element lying between the repetitive region and the *12S rRNA*.

The kDNA maxicircles of *T. cruzi *strains CL Brener and Esmeraldo have been assembled from whole-genome shotgun sequences (Fig. 1). Contigs assembled from the selected reads were merged to generate the maxicircle assembly covering the coding region, from which other contigs were added progressively based on mate pair information and identity to extend the assembly into the non-coding region. Consensus sequences were generated from assembled contigs with an average of 32X and 60X coverage for the CL Brener and Esmeraldo maxicircles, respectively. Assembly of both maxicircles was complicated by the presence of multiple variants due to apparent intrastrain heterogeneity of the maxicircle population. This variation was observed mainly in the non-coding region 4–6 kb upstream of the *12S rRNA*. Due to the difficulty in assembly of the non-coding region, the total lengths of the maxicircles were estimated based on coverage values of the coding region, assuming relatively equal coverage throughout the entire maxicircle and a population of maxicircles of equal size. Calculations based on these assumptions produce lengths of approximately 22 kb and 28 kb for CL Brener and Esmeraldo, respectively. Our consensus CL Brener and Esmeraldo maxicircles represent one variant for each of the non-coding region, and alternative assemblies with different variants were also assembled [see Additional file 1] (sequences available upon request). The differential size of Esmeraldo compared to CL Brener results from a larger non-coding region with larger duplicated conserved elements within the variable region, while coding region size is comparable. Polymorphisms in the non-coding regions likely represent natural variation within the multi-copy maxicircle population within each kinetoplast and among cells in culture, similar to the variation seen amongst multi-copy nuclear gene arrays [43].

The annotation of gene positions and lengths in the two *T. cruzi *maxicircles based on annotation of *T. brucei *and *L. tarentolae *is listed in Table 1. The order of the rRNAs and genes on the *T. cruzi *maxicircle transcripts is syntenic with the *T. brucei *and *L. tarentolae *maxicircles. A 5736-nt fragment of the maxicircle coding region [GenBank: U43567] that was cloned and sequenced from the Tulahuen (DTU IIe) strain [37] shows 98% and 88% identity to CL Brener and Esmeraldo maxicircles over the same region. The boundaries of the C-rich region (CR) 3 gene in *L. tarentolae *has been determined [44], but is not yet known for *T. brucei*; as the *L. tarentolae *sequence is divergent from *T. brucei *and *T. cruzi*, our annotation of this gene may be imprecise. Maxicircle-encoded gRNAs are likely present in *T. cruzi*, but not yet included in the annotation, awaiting validation of actual RNA editing events.

**Table 1:** Gene positions and lengths on CL Brener and Esmeraldo maxicircle consensus sequences

		CL Brener	Esmeraldo	CL BrenerEsmeraldo	
GENE	Editing of mRNA	position	position	Length	Length
12S rRNA		1–1161	1–1153	1161	1153
9S rRNA		1200–1808	1197–1804	608	607
ND8	Pan-edited	1853–2131	1857–2127	279	271
ND9 (revcomp)	Pan-edited	2195–2532	2192–2545	338	354
MURF5 (revcomp)	5' end edited *	2568–2715	2576–2722	148	147
ND7	Pan-edited	2857–3611	2862–3617	755	756
COIII	Pan-edited	3678–4100	3685–4109	424	425
Cyb	5' end edited	4175–5254	4167–5246	1080	1080
ATPase6	5' half edited	5292–5627	5288–5622	336	335
MURF1 (revcomp)	5' end edited	5675–7015	5681–7029	1340	1347
CR3	Pan-edited	7002–7120 **	7016–7136 **	uncertain	uncertain
ND1 (revcomp)	5' end edited	7116–8057	7132–8073	942	942
COII	Internal editing +4Us	8071–8699	8088–8716	629	629
MURF2	5' end edited	8725–9780	8750–9794	1056	1045
COI (revcomp)	Not edited	9771–11420	9785–11434	1650	1650
CR4 (revcomp)	Pan-edited	11471–11677	11487–11658	207	172
ND4	Not edited	11782–13095	11659–12872	1314	1214
ND3 (revcomp)	Pan-edited	13087–13279	12864–13051	193	188
RPS12	Pan-edited	13357–13547	13129–13315	191	187
ND5	Not edited	13568–15337	13335–15105	1770	1771

(revcomp) indicates reverse complemented genes encoded on the opposite strand

Gene positions are given relative to the start of the 12S rRNA

* MURF5 start position is uncertain, so 5' end editing is assumed to create the start codon

** CR3 5' and 3' end positions are uncertain, editing pattern, start, stop codons are unknown

The coverage of these genomes provides a high level of confidence in the assembled products. The CL Brener nuclear genome was sequenced to approximately 7X coverage, and Esmeraldo to 2.2X coverage. Despite this difference, the maxicircle genome coverage is deep for both strains. The high relative abundance of maxicircle sequences in the Esmeraldo dataset suggests that the Esmeraldo total cell DNA preparation had a higher content of kDNA relative to the CL Brener starting material. The difference was probably due to loss of intact network during phenol extraction, and not representative of the relative amount of kDNA in each strain.

### Nucleotide skew identifies gene orientation and editing locations

The *T. cruzi *maxicircle coding regions provide the primary templates for the RNA editing process. Using the maxicircle annotation for *L. tarentolae *and *T. brucei *the identification of ORFs and 'cryptic' genes that are extensively edited at the RNA level was straightforward. In comparative analyses CL Brener was used as the *T. cruzi *reference sequence.

**Figure 2:**
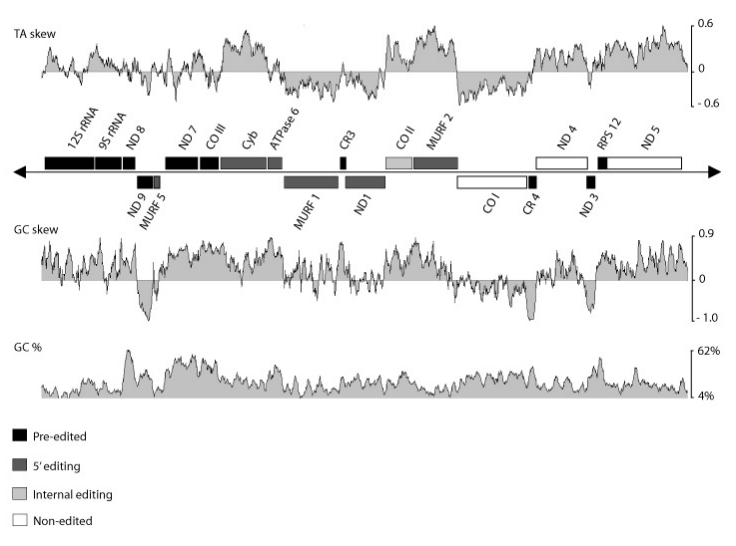
**Strong nucleotide biases in the coding region correlate with positions of unedited and pre-edited genes. **TA and GC skew analyses reflect the T-richness in protein-coding genes requiring little or no editing and G-richness in genes requiring extensive post-transcriptional RNA editing to create their coding regions. Window size = 100 bp.

The nucleotide frequency and skew throughout the *T. cruzi *maxicircle coding region correlated with gene orientation and extent of editing. The coding region is 74% AT-rich (Table 2), reflecting the AT-richness of the mitochondrial genome as a whole [45]. The AT-skew plot demonstrates the bias towards a T-rich coding strand that is more pronounced in non-edited genes (Fig. 2), as was also the case for *T. brucei *and *L. tarentolae *maxicircle genes [46]. The coding regions of extensively edited genes, which are comprised primarily of purines and have as much as half of the final edited mRNA content contributed by inserted uridines, display low %TA. The non-edited genes produce U-rich mRNAs similar in nucleotide content to extensively-edited mRNAs following editing; their U-richness is encoded in the DNA, lending support to the hypothesis that these genes were originally smaller, extensively-edited genes that were replaced by reverse-transcription of fully-edited versions by a retrotransposition event [46]. The GC skew plot illustrated a similar bias toward G-richness of the coding strand that is most pronounced in genes requiring extensive editing (Fig. 2). The observed GC skew resulted in the original naming as G-rich or C-rich regions [47]. The skew is much greater in the coding region than for the maxicircle overall (Table 2). The genes contain few C residues, suggesting a negative selection by the editing process that maintains cytosines only in regions where protein function and codon usage dictate their necessity. Regions of extreme GC bias as represented by the peaks in the %GC plot corresponded to extensively edited genes referred to as C-rich (CR#) or G-rich (G#) regions in *T. brucei *and *L. tarentolae*.

**Table 2:** Nucleotide composition of *T. cruzi *maxicircle regions

	Coding Region				Non-coding Region				Overall			
	CLB	Esmo	Tb	Lt	CLB	Esmo	Tb	Lt	CLB	Esmo	Tb	Lt

% A	38	37	37	35	46	52	59	52	39	40	44	38
% C	11	11	10	10	12	14	9	9	11	11	9	10
% G	15	15	16	13	10	6	8	6	14	13	13	12
% T	37	37	37	43	33	28	24	33	36	36	34	41
% A+T	74	74	74	77	79	80	83	85	75	76	78	79
% G+C	26	26	26	23	21	20	17	15	25	24	22	21
AT skew	0.01	-0.01	0.00	-0.10	0.17	0.30	0.43	0.23	0.04	0.04	0.14	-0.03
GC skew	0.15	0.16	0.24	0.15	-0.08	-0.38	-0.02	-0.17	0.10	0.05	0.19	0.10

Coding region region is defined as the contiguous nucleotide sequence of the maxicircle consensus from the start of *12S rRNA *through the end of the ND5 gene including intergenic regions, as defined for *T. cruzi *in Table 1. CLB, Esmo, Tb, and Lt represent *T. cruzi *CL Brener strain, *T. cruzi *Esmeraldo strain, *T. brucei *maxicircle coding sequence [GenBank:M94286] and variant region sequence [GenBank:Z15118], and *L. tarentolae *maxicircle sequence [GenBank:M10126]

### Similarity of RNA editing in *T. cruzi *and *T. brucei*

The nucleotide skew analyses indicated that *T. cruzi *maxicircle coding regions have GC skew patterns suggestive of extensive editing of genes that are differentially edited in *T. brucei *and *L. tarentolae*. Direct comparisons of the maxicircle genomes were performed to confirm this observation.

**Figure 3:**
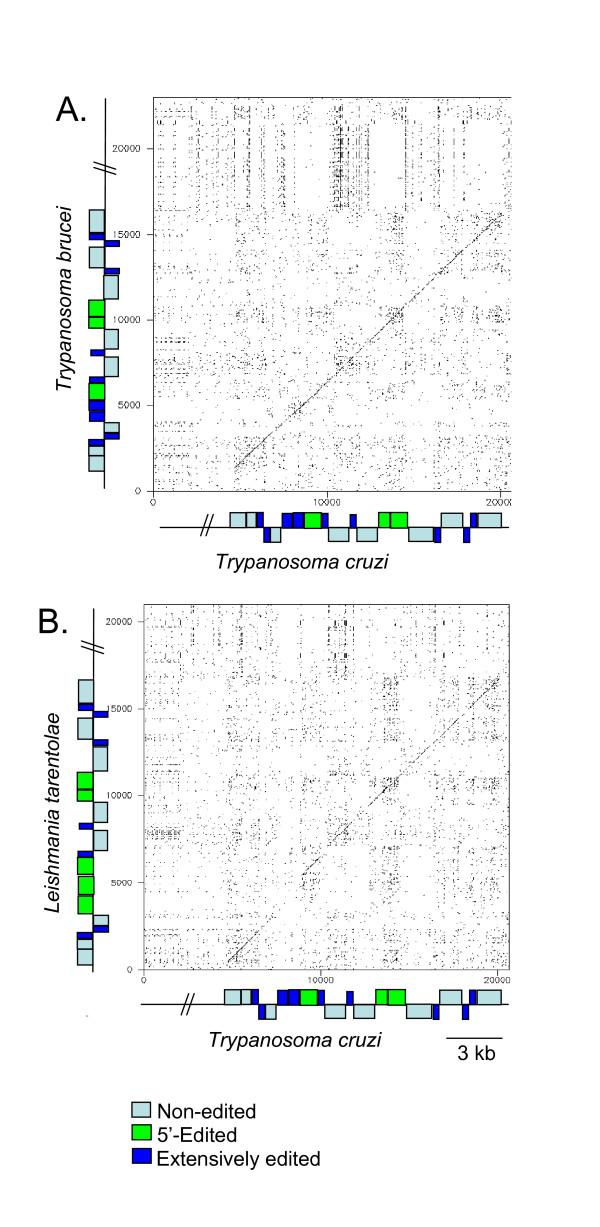
***T. cruzi *maxicircle genes are more similar to *T. brucei *than to *L. tarentolae*. **Dot plot analyses generated using Dottup comparing A) CL Brener and *T. brucei*, and B) CL Brener and *L. tarentolae *maxicircles. Dots represent an exact match of 10 bp.

Dot plot analyses of the CL Brener maxicircle with *T. brucei *or *L. tarentolae *identified regions of sequence homology between the two genomes (Fig. 3). Each dot represents 100% identity over a 12-bp window. The formation of diagonal lines running through the plots indicated extended areas of high sequence identity. The diagonals were limited to the protein-coding regions, with breaks in the *T. cruzi *vs. *L. tarentolae *plot corresponding to genes that are edited to different extents at the RNA level (*ND7*, *COIII*, and *ATPase6*) [48]. *T. brucei *showed greater overall sequence identity, represented by a diagonal line, matching CL Brener throughout the coding region; thus *T. cruzi *exhibited primary sequences suggestive of the more enthusiastic use of the RNA editing process as seen in *T. brucei*.

The sequences of the rRNAs and coding regions of pan-edited and non-edited genes of the *T. cruzi*, *T. brucei*, and *L. tarentolae *maxicircles were aligned and percent identity determined. The extensively edited genes have lower percent identity levels on average than the non-edited genes, while rRNAs showed the highest identities (Table 3). In comparisons of *T. cruzi *to *L. tarentolae *the identity jumps from 42% for edited genes to 76% for non-edited genes, indicating that RNA editing permits a greater genetic divergence at the level of the DNA template between genera. Identities for the rRNAs and the non-edited genes do not differ greatly, supporting this thesis. However, alignment of edited RNAs for *L. tarentolae *and *T. brucei *showed that differences at the DNA level are decreased following editing, providing higher percent identity in the final products [49]. The *T. cruzi *maxicircle sequence indicated that *ND8*, *ND9*, *ND7*, *COIII*, *ATPase6*, *G3*, *G4*, *G5*, and *RPS12 *are edited extensively as in *T. brucei*. Potential *T. cruzi*-specific regions for editing are presented below.

**Table 3:** Average percent identities among CL Brener, Esmeraldo,*T. brucei *and *L. tarentolae *rRNAs, gene coding regions and inferred protein sequences

		Edited	Non-edited	Non-edited	Entire coding
Comparison	rRNAs	Genes	Genes	Proteins	region
CL Brener vs. Esmeraldo	92.6%	86.2%	89.9%	81.7%	88.2%
CL Brener vs. *T. brucei*	79.6%	57.1%	77.8%	79.3%	73.3%
Esmeraldo vs. *T. brucei*	79.8%	55.2%	78.5%	81.0%**	72.5%
CL Brener vs. *L. tarentolae*	78.6%	41.8%*	75.4%	76.0%	65.2%
Esmeraldo vs. *L. tarentoae*	78.9%	41.6%*	76.0%	67.5%	64.7%
*T. brucei *vs. *L. tarentolae*	78.0%	42.4%*	76.6%	76.4%	64.7%

Non-edited genes include *ND5*, *ND4*,*COI*,*COII*,*ND1*,*MURF1*,*MURF2*,*Cyb*

Extensively-edited genes include *COIII*, *ATPase6*, *ND7*, *ND8*, *ND9*, *CR4*, *CR5*, *RPS12*

MURF5 and CR3 were not included because both 5'- and 3'-gene boundaries are uncertain

* *COIII*, *ND7 *and *ATPase6 *were not included in *Lt *comparisons due to different editing patterns

** *ND5 *not included due to frameshifts in Esmeraldo

Entire coding region includes contiguous sequence from the start of *12S rRNA *to the end of *ND5*

### A conserved element in the variable region of the non-coding sequences

Assembly of the CL Brener and Esmeraldo maxicircles allowed us to examine intra-specific variation at the genomic level. As the mitochondrial DNA of vertebrates is an excellent marker for intra-specific differences, the *T. cruzi *maxicircle is an interesting candidate for distinction between DTUs, and has been used previously to distinguish three clades [38,40]. Sequence differences were found in the gene-coding and non-coding regions. We begin with consideration of the non-coding regions where the sequences are most divergent.

**Figure 4:**
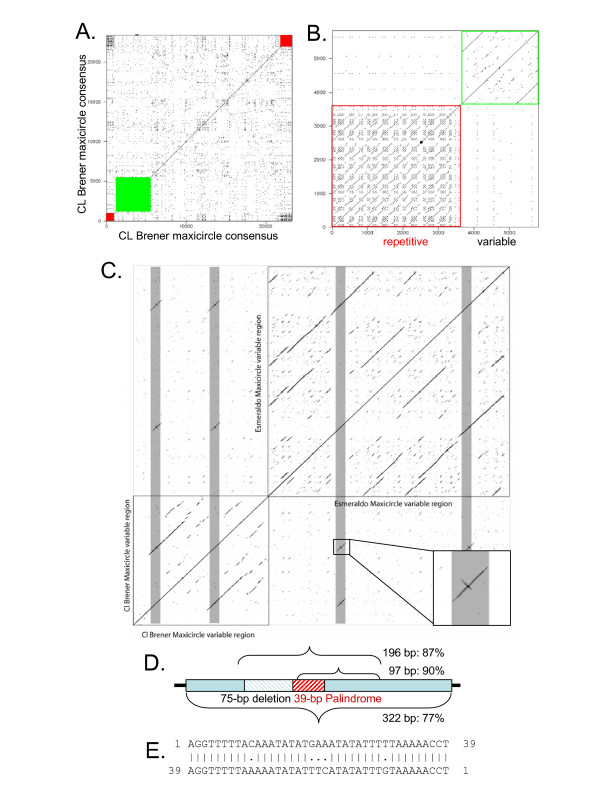
**The *T. cruzi *maxicircle non-coding region contains a variable and a repetitive region. **A) Dot plot analyses generated using Dottup comparing CL Brener maxicircle consensus vs. itself showing a repetitive region of short motifs (red) and a longer duplicated conserved element in the variable region (green). B) Expanded view of the repetitive non-coding region of the CL Brener dotplot. C) Dot plot comparison generated using Dotter software of CL Brener and Esmeraldo variable regions shows the presence of a conserved element (gray bars). For this analysis the CL Brener and Esmeraldo sequences of interest were joined for a combined comparison to self and to each other. Enlarged inset shows a conserved element with cross-structure indicative of a palindrome. D) The 39-bp imperfect palindrome within the variable region conserved element. E) Schematic representation of percent identity of variable region conserved element among all CL Brener and Esmeraldo assemblies.

The non-coding region differed significantly between CL Brener and Esmeraldo maxicircle assemblies and could be divided into regions that we refer to as 'variable' and 'repetitive' (Figs. 1, 4A, B). The variable region was defined because several variants were assembled in CL Brener and Esmeraldo, with most of the variation occurring within 4–6 kb upstream of the coding region [see Additional file 2]. A sequence element represented at least twice per maxicircle is conserved between the CL Brener and Esmeraldo maxicircles in this region (Fig. 4C). The core of the element was 97 bp with an average of 90% identity, dropping to an average of 87% over 195 bp and 77% over 325 bp among all variant contigs assembled for the two strains (Fig. 4D) [see Additional file 3]. The cross structure present within the conserved element is due to a match to the reverse complement of a 39-bp sequence that forms an imperfect palindrome near the beginning of the conserved 97-bp core (Fig. 4E). CL Brener has at least two imperfect copies of the element, of which the 3' copy has a 75-bp deletion in the 195-bp area. Interestingly, this deletion removes the first six bases of the 39-bp palindrome. The two 97-bp cores present in the consensus sequences reside 2237 nt and 1085 nt upstream of the *12S rRNA *in CL Brener, and 3346 nt and 811 nt upstream of the *12S rRNA *in Esmeraldo. Our consensus assembly may under-represent the size of the variable region, meaning that a single maxicircle may contain more than two copies of this element. The presence of duplicated conserved elements in this region combined with intrastrain variability precluded conclusive consensus determination. Alternative assemblies of this variable region produced catenated, tandem repeats with multiple copies of the conserved elements that are likely assembly artifacts not representative of an actual molecule [see Additional file 1].

Conservation of the 325-bp element between CL Brener and Esmeraldo within the otherwise heterogeneous non-coding region implies a conserved function. A search for similar elements within the variable regions of *T. brucei *and *L. tarentolae *maxicircles, their entire genomes, and the NCBI databases, yielded no match. However, the element is in the same region upstream of the *12S rRNA *and shows a tandem organization similar to a duplicated sequence described in the Variable Region III of the *T. brucei *maxicircle [50]. Of the two copies in *T. brucei*, the 5' copy is longer, while the 3' copy is shorter, analogous to the pattern in CL Brener. No sequence identity can be seen between the *T. cruzi *element and the *T. brucei *sequence, but the common organization within the non-coding regions is suggestive. The 39-bp palindrome sequence found in *T. cruzi *is an attractive candidate for a dimeric or multimeric protein complex binding site. A search for palindromes within the analogous tandem repeats of *T. brucei *revealed a 14-bp palindrome (TAAATTTAAATTTA) present in both repeats. The *T. brucei *palindrome is shorter and of different sequence composition relative to the *T. cruzi *palindrome.

The repetitive portion of the non-coding region was visualized as a dense block of identity in dot plot analyses comparing this region to itself (Fig. 4A). In contrast to the variable region, the repetitive region showed no conservation between CL Brener and Esmeraldo, with unique repeated motifs of different size and sequence composition for each strain. Small motifs were conserved within the repeated blocks of sequence for each strain [see Additional file 4]. The CL Brener repetitive region is composed of variants of 267, 330, or 390 bp. Esmeraldo showed greater intrastrain variability with repeated elements of 296, 307, or 396 bp.

Previous comparisons of repetitive region motifs found no conservation between *T. brucei *and *L. tarentolae *[2]. *T. cruzi *also shows no conservation of motifs between strains or among trypanosomatid species. All are A-rich with many tandem poly (A)-tracts and a greater than average AT skew (Table 2). Nucleotide composition analysis revealed a higher %AT in the repetitive region than the coding region or the overall maxicircle sequence for all trypanosomatid maxicircle sequences (Table 2). The variability of these repeats may be useful as specific strain or DTU markers.

### Variability in the *T. cruzi *coding regions

Protein-coding regions provide specific markers for the variation among *T. cruzi *strains [38,40] and, in contrast to the non-coding regions, they have a recognizable selective pressure. In considering the functional utility of the maxicircle as a molecular marker for strain identification and classification, the coding regions present variation that is likely conserved among DTUs or subgroups of strains, while the variable regions may allow differentiation among closely related isolates. No significant variation was observed within the consensus assembly of the coding region of each strain, indicating high intra-strain homogeneity of the maxicircle population. The coding region showed high levels of identity between the two *T. cruzi *strains, with some 'editable' differences observed at SNPs and indels in poly (T) tracts within edited genes. Comparisons of the coding regions from CL Brener and Esmeraldo showed identity ranging from 86.2% for extensively edited genes, to 89.9% for non-edited genes, to 92.6% for rRNAs (Table 3). Several examples of small indels as well as a substantial deletion within the coding region of the maxicircle with potential detrimental effects on protein translation will be described.

### Truncation of two Esmeraldo maxicircle genes

**Figure 5:**
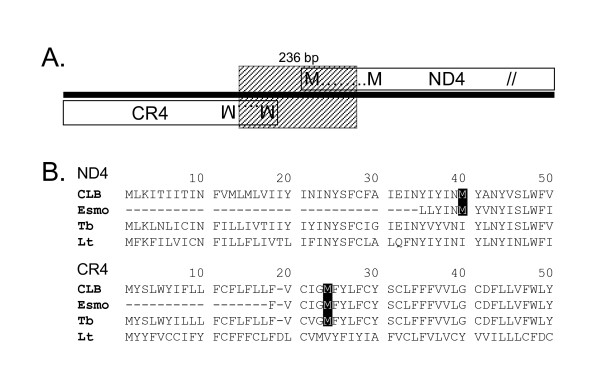
**Esmeraldo has a strain-specific deletion truncating the 5' ends of two genes. **A) Schematic representation of the 236-bp deletion in Esmeraldo (34 nt of CR4, 105 nt of intergenic region, and 98 nt of ND4). B) Alignments of the N-terminus of predicted proteins from *T. cruzi *strains CL Brener and Esmeraldo, *T. brucei *and *L. tarentolae*. Conserved, alternative downstream start-site methionines are boxed.

The most pronounced coding-region difference between the two strains was a 236-nt deletion in Esmeraldo relative to the CL Brener maxicircle, resulting in 5'-end truncations of C-rich region 4 (*CR4*) and NADH dehydrogenase subunit 4 (*ND4*) (Fig. 5A). These two genes are transcribed on opposing strands in a head-to-head orientation, and the deletion eliminates the predicted initiation codons for both genes. Esmeraldo *ND4 *lacked the first 99 nt of its coding region relative to CL Brener (Fig. 5B). A downstream in-frame AUG may allow for the expression of a truncated protein of 397 aa, compared to the 437-aa CL Brener protein. The deletion leaves 17 nt of the former reading frame upstream of the potential start codon. The transmembrane prediction program TMpred [51] indicated that the first two membrane spanning regions of ND4 to lie at aa 4–21 and aa 45–64, thus the deletion eliminates the first predicted transmembrane domain.

The Esmeraldo *CR4 *is missing the first 34 nt of its coding region as defined in CL Brener (Fig. 5B). By analogy with the extensively edited *T. brucei CR4*, 50 nt of the edited ORF would be lost. An in-frame AUG, 15 nt downstream of the deletion, may allow for translation of a truncated protein of 123 aa, compared to 147 aa predicted for CL Brener. TMpred predicts the first two transmembrane domains of CR4 to be at aa 4–28 and aa 32–55, thus the truncated CR4 protein would lack the first transmembrane domain. The function of *CR4 *is unknown, but appears conserved with the *T. brucei *and *L. tarentolae *sequences that display 77% and 74% identity at the edited mRNA and predicted protein levels, respectively.

A BLAST search using the corresponding region from CL Brener confirmed that the deleted sequence is not present in any of the Esmeraldo reads; greater than 50X regional coverage gives high confidence that the deletion is found in all Esmeraldo maxicircles. To query directly the absence of this region in Esmeraldo, we examined Esmeraldo DNA along with six additional DTU IIb strains and 13 strains representing the five other DTUs (data not shown). PCR using primers flanking the Esmeraldo deletion showed that only Esmeraldo had this deletion, and no vestige of a full-length version was detected; neither of the DTU I representatives yielded amplification products, likely due to SNP variation in the primer annealing site.

The unique appearance of this deletion in the Esmeraldo strain suggests that it may be a recent event tolerated in a cultured strain, but deleterious in wild populations. The indels detailed in the following section point to additional anomalies in the Esmeraldo maxicircle that may be incompatible with virulence of this strain.

### ND5, MURF1, and MURF2 frameshifts

**Figure 6:**
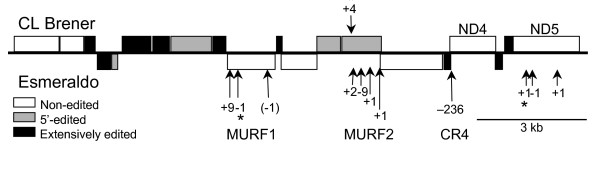
**Summary of CL Brener and Esmeraldo coding region polymorphisms. **Specific indels lying in regions not processed by RNA editing in *T. brucei *are indicated for each strain. Indels resulting in the creation of a downstream termination codon are highlighted by an asterisk (*).

Multiple additional small indels ranging from 1 to 9 bp were found in both *T. cruzi *strains in areas of genes not requiring RNA editing in *T. brucei *or *L. tarentolae *(Fig. 6), with the preponderance appearing in Esmeraldo. None of the indels were shared between the strains, several of which result in the creation of premature termination codons.

The Esmeraldo *ND5*, which is not edited in *T. brucei *and seemingly does not require editing in CL Brener, has three frameshift mutations relative to the CL Brener sequence [see Additional file 5] The first is an insertion at position 479 resulting in the introduction of two in-frame stop codons 14- and 34-nt downstream. The reading frame is restored by a single nt deletion relative to the CL Brener sequence at position 514. Another single nt deletion at position 1210 again results in a frameshift that alters the remainder of the ORF. Thus Esmeraldo *ND5 *translated directly from the DNA sequence would generate a truncated protein of 165 aa compared to the 589 aa product of CL Brener.

*MURF1 *is not well annotated in other species. 5' editing could create the initiation codon, but no analogous start codon is annotated in *T. brucei *or *L. tarentolae*. We have used the largest continuous ORF present in *T. brucei *to represent the start point for this gene. Using the equivalent start position in *T. cruzi*, the CL Brener *MURF1 *codes for a 446 aa protein. Esmeraldo *MURF1 *contains two single-nt deletions at positions 73 and 1225 relative to the CL Brener sequence, both of which result in internal frameshifts [see Additional file 5]. In addition, Esmeraldo shows a 9-bp insertion at positions 1315–1323. The deletion at position 73 may have no effect, as the 5' end of the ORF is undetermined. The frameshift at position 1225 introduces an in-frame stop codon that would result in a product truncated by 35 aa relative to CL Brener.

The *T. brucei MURF2 *mRNA is edited at the 5' end by the insertion of 24 uridine residues to create the initiation codon and the reading frame up to nt 45 of the ORF. This localized editing appears to be conserved in *T. cruzi*; a conserved amino terminus can be predicted after hypothetical RNA editing in both strains. However, CL Brener and Esmeraldo both show frameshift mutations in areas that are apparently not edited in *T. brucei *[see Additional file 5]. The CL Brener *MURF2 *had a 4-nt insertion relative to *T. brucei *after nt 266 of the predicted edited ORF, creating in a frameshift that introduces a premature stop codon. Thus a 101-aa protein would result, containing the first 88 of 357 aa in common with *T. brucei *and *L. tarentolae*. The Esmeraldo *MURF2 *contained four insertion or deletion events relative to the *T. brucei *sequence: a 2-nt insertion at position 322, a 9-nt deletion at 493, and two single-nt deletions at positions 746 and 770. Cumulatively, these differences result in a frameshift starting at aa 181 and continuing through aa 249, where the frame reverts to the *T. brucei *cadence, leaving 69 aa in the central portion of the 357-aa protein altered. Approximately half of amino acids in this shifted region are conservative changes relative to CL Brener.

## Discussion

We report the complete maxicircle consensus sequences from two strains of *T. cruzi *that are syntenic with the coding regions of maxicircles in *T. brucei *and *L. tarentolae*. All three mitochondrial genomes contain 18 protein-coding genes and two rRNAs. Strain-specific frame shifts were documented in several *T. cruzi *genes at positions not edited in other kinetoplastids. Comparison of the non-coding region of the two *T. cruzi *strains showed a duplicated conserved element containing an imperfect 39-bp palindrome located in the variable region, and divergent repetitive motifs of similar nucleotide composition in the repetitive region. Several variants of the CL Brener and Esmeraldo non-coding region were assembled, indicating that maxicircle sequence and size are heterogeneous in these strains. The size of the total assemblies differed between CL Brener and Esmeraldo strains due to differences in the repetitive area of the non-coding region; a strain-specific 236-nt deletion was found in the Esmeraldo coding region. These genomes represent two of the three *T. cruzi *maxicircle clades defined previously [38,40].

The profound influence of RNA editing on maxicircle genes overshadows all other processes affecting mitochondrial genomes. The main evolutionary forces shaping the nucleotide composition and skew of metazoan mitochondrial genomes are postulated to be the asymmetric processes of DNA replication and RNA transcription, biasing nucleotide frequency due to differential mutation and selection pressures [52]. While these processes may have some effect, RNA editing is the dominant influence affecting AT and GC skew on both strands of the coding region. Unlike metazoans, where all protein-coding genes are encoded on the same strand, maxicircle genes are transcribed as polycistronic transcripts from both strands [47,53], thus the influence of coding bias and directional mutational bias from transcription would be negated.

Generally, protozoan mitochondrial genomes display lower %GC than mitochondrial genomes from metazoans, except for those of insects [45], with the trypanosomatid mitochondria showing among the lowest %GC of all protozoans (Table 2). The extreme AT-richness of trypanosomatid mitochondrial genomes is due to the repetitive portion of the non-coding region (Table 2) that accounts for approximately 15–18% of the *T. cruzi *maxicircle sequence and 17–30% of the entire maxicircle sequence in various *T. brucei *strains [50]. AT skew is strong in the non-coding region of the maxicircle, but not in the coding region, while GC skew is inverted. Different directional biases affect mutation rates in these two regions, related to gene content issues in the coding region and to potential structural and functional constraints on the non-coding region for kinetoplast organization, replication and division.

The poly (A) tracts of the repetitive region may induce bending of the DNA over long stretches. DNA bending was first discovered in minicircles [54], and is found in multiple eukaryotic and prokaryotic DNAs, especially in control regions such as promoters [55] and origins of replication [56]. In minicircles from many kinetoplastids, *T. cruzi *being an exception, bends are located adjacent to conserved sequence blocks that are likely binding sites for kinetoplast replication proteins [57]. The bent region may serve a topological function for packing of the minicircles into the characteristic disc structure [58]. Maxicircles in *T. equiperdum *are concatenated in a network independent of minicircles, linked together at a protease-resistant core [59]. The bent nature of the repetitive region may form a solenoid structure that intertwines the maxicircles within this central core.

An intriguing conserved sequence element of ~300 bp is present in equivalent positions of the variable region of the *T. cruzi *CL Brener and Esmeraldo strains. A 39-bp imperfect palindrome within this element may serve as a specific recognition sequence for DNA binding proteins. Palindromes may serve as binding sites for dimeric or multimeric proteins at promoter regions and replication origins. Origins of replication have been mapped to the non-coding region of *T. brucei *and *C. fasciculata *[49]. The *T. brucei *origin lies in the variable region just upstream of the *12S rRNA*; two conserved 600-bp tandem repeats were found in the *T. brucei *maxicircle assembly [41,50]. The *T. cruzi *and *T. brucei *elements show no conservation at the sequence level, however the position and tandem arrangements suggest a common function. In mammalian mitochondria transcription initiation and DNA replication begin at the same site just upstream of the rRNA genes [51]. In *T. brucei *the *12S rRNA *primary transcript initiates approximately 1.2 kb upstream of the gene [52], in the same region as the origin of replication. This variable region element may have a dual function as both replication origin and promoter.

**Figure 7:**
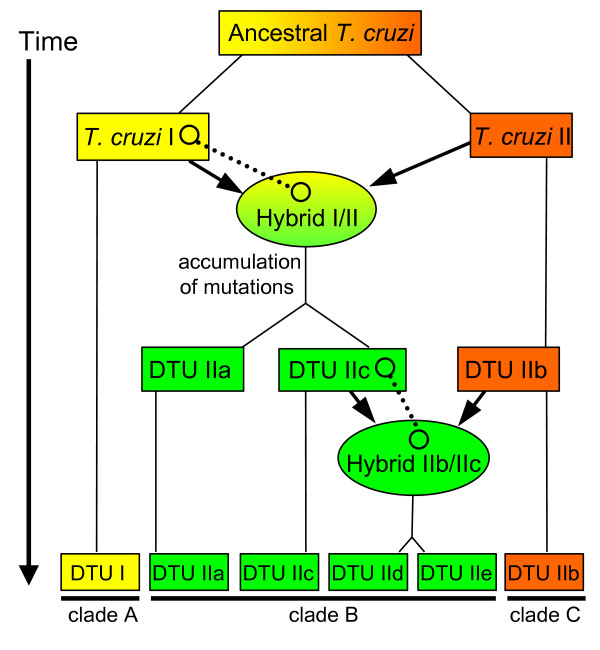
**Postulated inheritance of maxicircle genomes superimposed upon the two hybridization events in *T. cruzi*. **The maxicircle clades defined by Machado and Ayala [31] are overlaid on the schema of the evolutionary history [23] of *T. cruzi *sub-groups. In agreement with the phylogenetic comparisons among the three clades, the maxicircle donor in the first hybridization event between the DTU I (clade A, yellow) and DTU IIb (clade C, orange) strains was the DTU I parent. Open circles and dotted lines represent maxicircle inheritance. Over time, the maxicircle in the new hybrid line accumulated a unique set of mutations distinguishing them from the DTU I parental maxicircle, designated clade B (green), as seen in sibling DTUs IIa and IIc. In the second hybridization event between strains from DTU IIc and DTU IIb, the clade B maxicircle was passed on to the progeny represented by DTUs IId and IIe. The sequences assembled for this manuscript represent maxicircles from clades B (CL Brener) and C (Esmeraldo).

Experimentally-generated hybrids in *T. brucei *indicate that inheritance of maxicircle sequences is uniparental, while minicircle inheritance is biparental [60]. *In vitro *crosses of differentially-tagged *T. cruzi *strains also displayed uniparental maxicircle inheritance [41]. Sequencing of a 1.5-kb region of the maxicircle from 45 *T. cruzi *strains covering *COII *and *ND1 *identified three clades encompassing the six defined DTUs [40]. Esmeraldo falls into clade C (DTU IIb), while CL Brener represents clade B (DTU IIa, IIc, IId, and IIe). The same pattern of three clades was observed in phylogenetic analysis of *CYb *[38]. Superimposing the maxicircle clades onto our minimalist reconstruction of *T. cruzi *hybridization events [25] (Figure 7), clade B likely evolved from a clade A (DTU I) maxicircle inherited in the common ancestor of DTU IIa/IIc strains following the prior DTU I/DTU IIb hybridization. The clade B maxicircle was then passed on to the DTU IId/IIe hybrids from their DTU IIc-type ancestor [38]. Bootstrap values from phylogenetic analysis of both *COII*-*ND1 *and *CYb *demonstrate the closer association of clade B with clade A than clade C, supporting the uniparental inheritance of DTU IIa/IIc hybrids maxicircles from a DTU I-like strain. Analysis of a DTU I maxicircle should confirm the original donor of the clade B maxicircle.

The mixing of parental minicircles observed in *T. brucei *hybrids [60] may be a method of ensuring preservation of the specific templates for RNA editing that are carried in the gRNA genes, countering loss of gRNAs through random segregation of the minicircles to daughter cells [61]. gRNAs from different strains might bear little resemblance to one-another at the sequence level, but their ability to direct the editing process may be indistinguishable relative to the mRNA. The physical mixing of the maxicircles during the fusion process may be inhibited by the structural organization of this relatively large molecule within the minicircle-clogged network. With the identification of multiple specific markers in the maxicircles of different strains, recombination or exchange during cellular fusion events may be detected. The spatial arrangement of the maxicircle within the kDNA network is not known, and the maxicircle genomes allow the design of specific probes to determine their organization within the kDNA disc.

Edited genes consistently show a lower percent identity than non-edited genes among the genera. This difference is minor between CL Brener and Esmeraldo, but increases in comparisons between the *Trypanosoma *species, and is greater still between the *Trypanosoma *spp. and *L. tarentolae *(Table 3). Editing thus corrects for genomic variability in the number and distribution of thymidine residues, allowing protein sequence conservation despite highly diverged genomic sequences. The *ND7*, *COIII*, and *ATPase6 *transcripts are extensively edited in *T. brucei *and *T. cruzi*, but only require 5' editing in *L. tarentolae *[48]. *ND8, ND9, ND3, CR3, CR4*, and *RPS12 *are extensively edited in all three species but the pre-edited genes are shorter in *T. brucei *and *T. cruzi *than in *L. tarentolae*. As such *Trypanosoma *spp. maintain a smaller maxicircle coding region, but require more gRNA information than *Leishmania. Trypanosoma *branches early in the evolution of trypanosomatids, before the *Leishmania *clade [62], and in accordance with this evolutionary schema the greater number of extensively-edited genes in the *Trypanosoma *may more closely resemble the ancestral state of the Trypanosomatina. The loss of extensive RNA editing seen in *Leishmania *would represent a derived state [48,57].

The pressure to maintain extensive amounts of RNA editing might be revealed by the consequences of the process. The size of the primary gene template encoded in the maxicircle is radically minimized, with up to half of a gene's coding information stored in gRNAs whose function is largely unaffected by transition mutations. Insertions and deletions of thymidines in the edited gene coding regions are also tolerated due to their subsequent reshuffling during RNA editing. RNA editing can compensate for certain DNA mutations and allow for production of wild-type functional proteins. RNA editing is likely to have evolved in a free-living ancestor of trypanosomatids that migrated between aerobic and anaerobic environments [63]. Thus protein functionality may be maintained despite mutation over many generations in the absence of selection, while the cells live in an environment that relieves them of the necessity to perform oxidative phosphorylation or other aerobic mitochondrial activities.

The *T. cruzi *strain-specific indels of Esmeraldo and CL Brener in gene coding regions not edited in *T. brucei *may represent mutations that are tolerated in a cultured strain, but prohibitive to completion of the digenic lifecycle, as is seen in the degenerate UC strain of *L. tarentolae *[44]. In short, they could represent laboratory-generated dead-ends. However, these mutations may give insight into evolution of the RNA editing process. The strong uridine bias evident in the non-edited genes suggests that all of the genes were at one time subject to extensive RNA editing. The persistence of 5'-end editing supports the theory that reverse transcriptase mediated recombination of partially edited intermediates eliminated the need for whole-ORF editing in several instances. Perhaps residual gRNAs for non-edited genes are maintained in the heterogeneous minicircle population due to the structure of the minicircles themselves in *T. cruzi *and *T. brucei*. While in *L. tarentolae *a single gRNA is contained on each minicircle, the *Trypanosoma *minicircles carry two to four genes. If maxicircle genes are under pressure to 'escape' from editing, the single-gRNA minicircles of *Leishmania *are more easily lost from the population than a gRNA in the triplet or quartet minicircle arrangements of *Trypanosoma *[64], effectively pushing *Leishmania *toward a more rapid loss of editing information. As such, the *Trypanosoma *spp. may carry an extra load in the form of gRNAs for genes that no longer require editing, but that could be utilized in the repair of indel mutations in normally unedited gene regions as a post-transcriptional proofreading mechanism. With the further characterization of the minicircle populations and RNA editing events of the CL Brener and Esmeraldo strains, these possibilities can be addressed directly.

## Conclusion

The complete assembly of two *T. cruzi *maxicircle genomes was performed using data generated by genome sequencing projects for the CL Brener and Esmeraldo strains. These sequences represent two of the three maxicircle mitochondrial genome clades of *T. cruzi*. The coding region of both maxicircles shows conservation of gene content and order with other kinetoplastids, with similar editing patterns conserved within the *Trypanosoma*. Strain-specific indels may indicate unique editing events or non-functional genes. The non-coding regions share duplicated, conserved elements of approximately 325 bp situated upstream of the ribosomal RNA genes that may serve as origins of replication and transcription initiation sites. Repetitive portions of the non-coding region are distinct for each strain, but display similarly poly-A rich sequences that may induce DNA bending and serve a structural function.

## Methods

The strategy employed in the construction of CL Brener and Esmeraldo maxicircles comprised the gathering and assembly of reads generated by the TIGR-SBRI-KI *T. cruzi *Sequencing Consortium (TSK-TSC) [42] with high identity as determined by BLAST to previously published maxicircle sequences: 5.7 Kb fragment of *T. cruzi *maxicircle, [GenBank:U43567], *T. brucei *maxicircle, [GenBank:M94286] and *L. tarentolae *maxicircle [GenBank:M10126]. Various assembly software packages were used in the assembly with equivalent results: Celera Assembler [65], Phrap [66] and SeqMan (Lazergene, DNAStar). All assembly software was executed using the default parameters. Additionally, any contigs generated by the *T. cruzi *genome consortium and having high identity to maxicircle sequences mentioned above were merged together. Other contigs were iteratively added to this initial sequence based on sequence identity and mate pair linkage. The iteration was finished when the generated sequence had similar patterns on its both ends, as evidence that a circular sequence was obtained. The sequence was dismantled and its contigs were submitted to scaffolding by Bambus software [67]. The final maxicircle sequence was obtained by manual overlapping and concatenation of Bambus scaffolds. Both strategies generated identical results for the coding region sequence, but only the latter had produced a putative circular sequence. Consensus maxicircle sequences for *T. cruzi *strains CL Brener and Esmeraldo are available through [GenBank:DQ343645 and GenBank:DQ343646], respectively.

Manual annotation of gene coding regions was performed by comparison to the published *T. brucei *maxicircle sequence, [GenBank:M94286]; the *L. tarentolae *maxicircle sequence, [GenBank:M10126]; *T. brucei *maxicircle variable region, [GenBank:Z15118]; and further annotation of *T. brucei *and *L. tarentolae *by Larry Simpson available through his Uridine-Insertion/Deletion Edited Sequence Database [12].

Linear annotated maps of the maxicircle sequence and TA skew, GC skew and GC percentage graphs of the coding region were created using Artemis [68]. Circular maps were generated using Bioedit [69]. Dotplot graphs of *T. cruzi *sequence plotted against *T. brucei *and *L. tarentolae *showing exact matches of 10-bp wordsize were generated by Dottup application from the EMBOSS software suite [70]. Dotplot graphs of CL Brener and Esmeraldo maxicircle variable regions plotted against themselves and each other were generated using the Dotter software package [71]. The Dotter software package uses greyscale to display less perfect matches as lighter shaded dots, and also displays matches to the reverse complement.

Alignments of the coding regions of the maxicircle genes and translated protein products of *T. cruzi*, *T. brucei*, and *L. tarentolae *were performed using ClustalX. Alignments were adjusted manually using BioEdit [69]. BioEdit was used to calculate nucleotide composition and percent identity matrices.

MEME/MAST software [72] was used to define and locate motifs within the repetitive region of the maxicircles. Multiple occurrences of each motif were aligned and submitted to Weblogo [73]. This software generates a graphical representation of multiple alignments where the character height represents the degree of conservation of each monomer.

## Authors' contributions

SW carried out sequence assembly and manual annotation, generated comparative genomic data and figures, and drafted the manuscript. GC carried out sequence assembly and generated comparative genomic data and figures. NE coordinated the genome sequence project that generated the original sequence data at TIGR and facilitated access to original sequence data and genomic analysis software for assembly and figures. BZ conceived of the study and provided the Esmeraldo strain genomic DNA used in the genome sequencing project. DC participated in data analysis and interpretation. NS conceived of the study, participated in data analysis and interpretation, and drafted the manuscript. All authors contributed to the editing of the text and approved the final manuscript.

## Supplementary Material

Additional File 1 Alternative assemblies of the maxicircle variable regions. Dotplots generated using Dottup of A) CL Brener and B) Esmeraldo maxicircle large variable region assemblies that represent artificial constructs show large duplicated regions. Click here for file

Additional File 2 Distribution of conserved motifs within variable region assemblies of Emeraldo and CL Brener maxicircles. Colored rectangles represent the conserved motifs defined by MEME on four representative alternative assemblies of the variable region. The positional frequency of each nucleotide in each motif generated using Weblogo is depicted below the figure. The 39-bp palindrome sequence is contained within motif 10. Motifs 5 (yellow) and 10 (light gray) depict the core of the conserved element across strains. Click here for file

Additional File 3 Conserved sequence elements of the maxicircle variable region. Alignment of conserved element sequence variants from CL Brener and Esmeraldo assemblies was generated by ClustalX. Consen indicates ClustalX consensus annotation. Click here for file

Additional File 4 CL Brener maxicircle repetitive region motifs. Schematic organization of motifs in CL Brener repetitive non-coding region defined using MEME analysis. Click here for file

Additional File 5 Strain-specific indels in non-edited genes resulting in frameshifts. ND5, MURF1 and MURF2 predicted protein alignments and partial coding region alignments displaying effects of indel mutations. Arrows indicate the position of indel mutations. Black highlighting indicates regions of frameshifts relative to *T. brucei*. Premature termination codons are underlined in DNA alignments and depicted as stars in protein sequences. MURF2 protein sequence is based on predicted 5' editing conserved with *T. brucei *with insertion of 24 U's up to nt 45. Click here for file
