# Supplementary figures and images for: Trypanosoma cruzi mitochondrial maxicircles display species- and strain-specific variation and a conserved element in the non-coding region
Source: BMC Genomics. 2006 Mar 22;7:60. doi: 10.1186/1471-2164-7-60 (PMC1559615; doi:10.1186/1471-2164-7-60)

## Slide 1
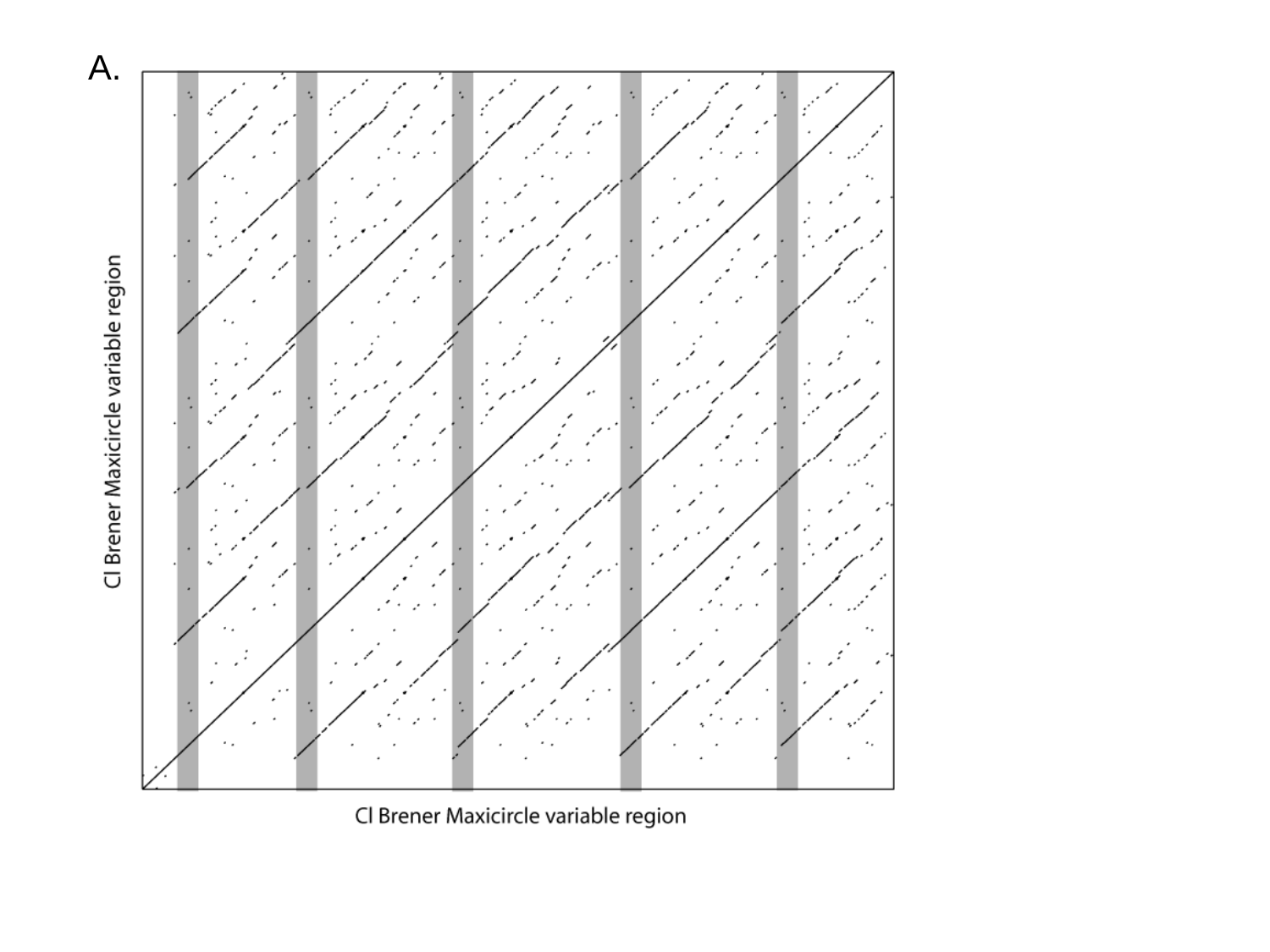

A.

## Slide 2
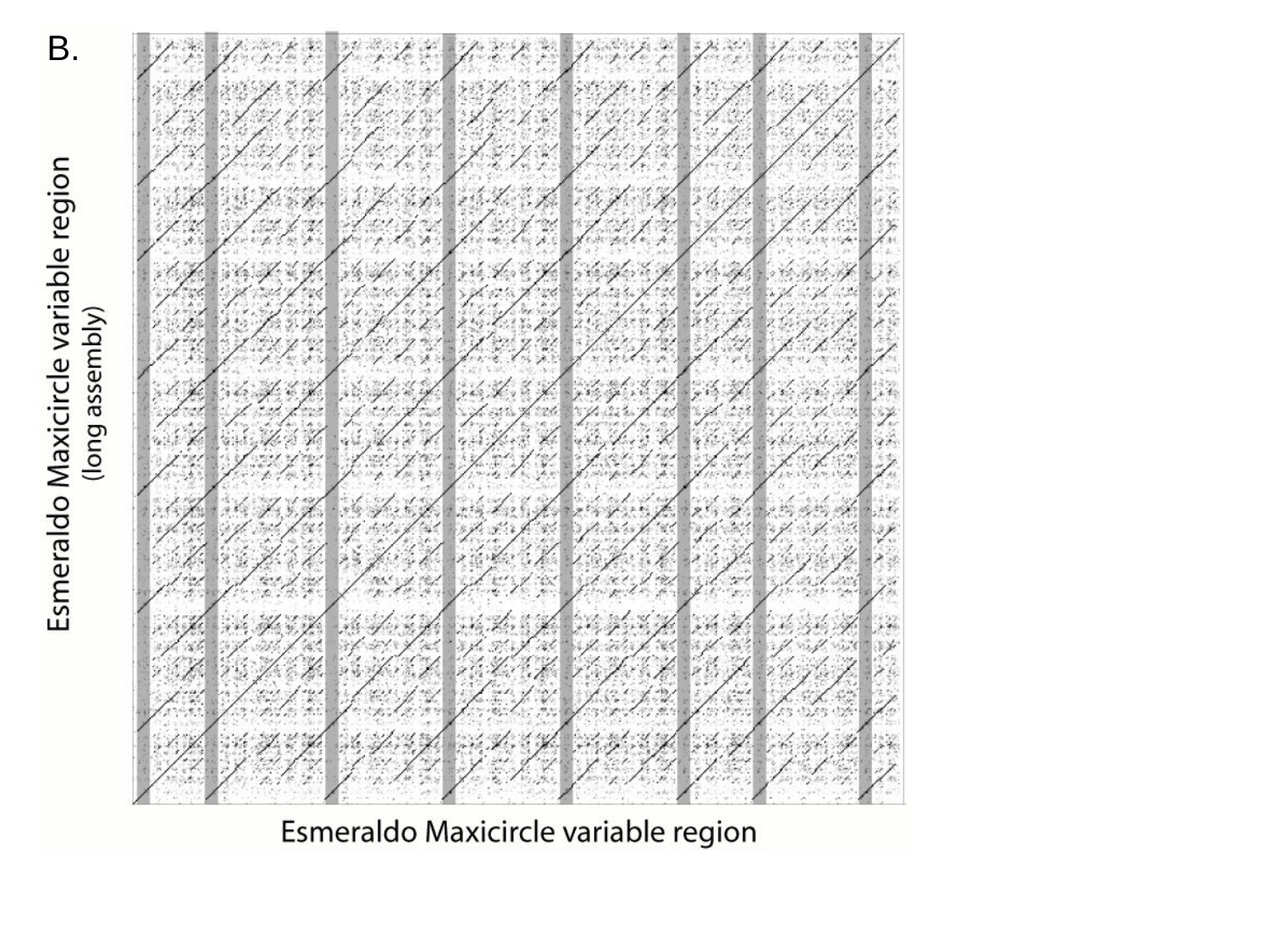

B.

Supplement: Additional File 1 — Alternative assemblies of the maxicircle variable regions. Dotplots generated using Dottup of A) CL Brener and B) Esmeraldo maxicircle large variable region assemblies that represent artificial constructs show large duplicated regions. [file 1471-2164-7-60-S1.ppt]

## Slide 1
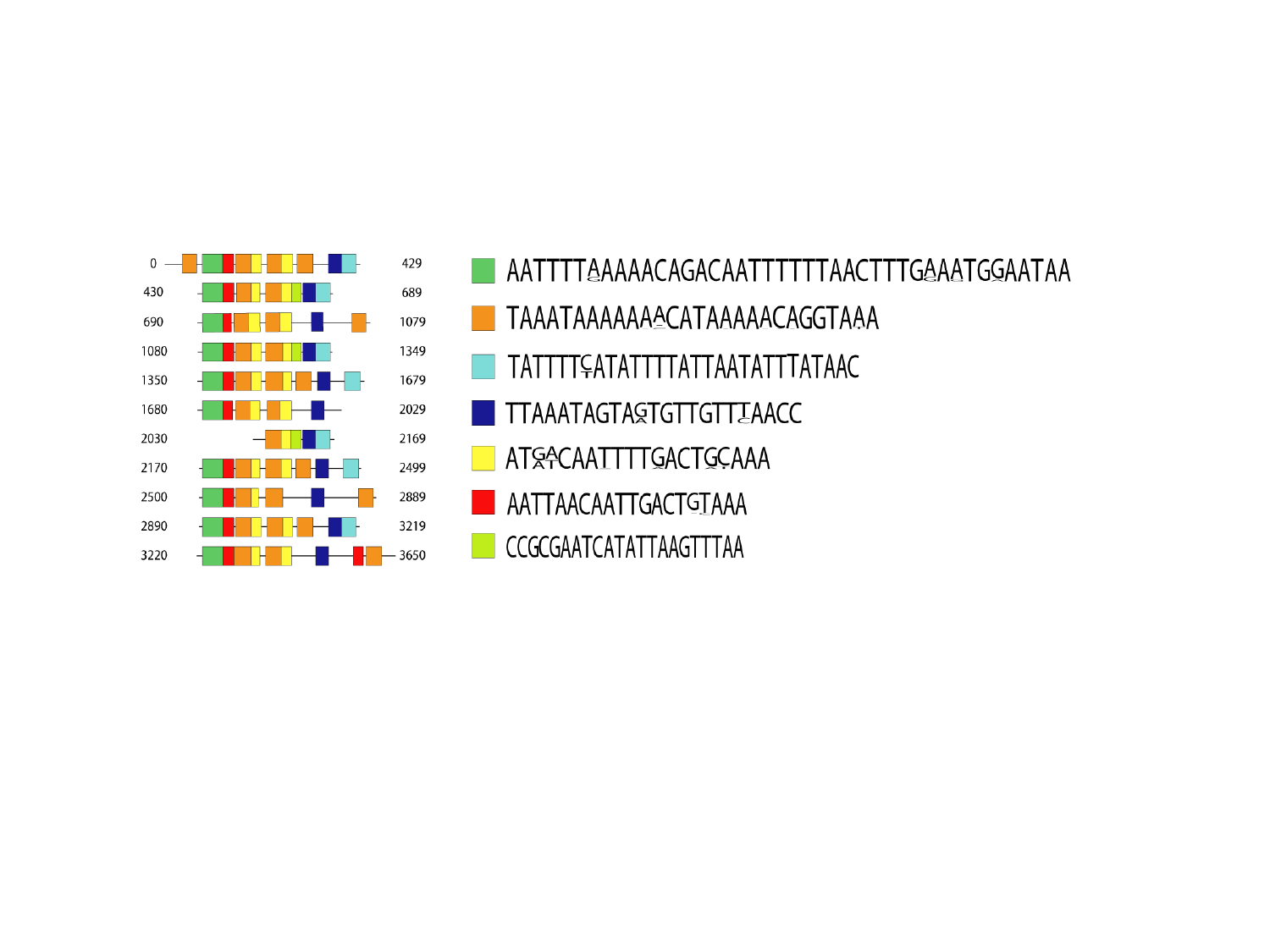

Supplement: Additional File 4 — CL Brener maxicircle repetitive region motifs. Schematic organization of motifs in CL Brener repetitive non-coding region defined using MEME analysis. [file 1471-2164-7-60-S4.ppt]
