# Supplementary material for: Trypanosoma cruzi mitochondrial maxicircles display species- and strain-specific variation and a conserved element in the non-coding region
Source: BMC Genomics. 2006 Mar 22;7:60. doi: 10.1186/1471-2164-7-60 (PMC1559615; doi:10.1186/1471-2164-7-60)
Supplement: Additional File 2 — Distribution of conserved motifs within variable region assemblies of Emeraldo and CL Brener maxicircles. Colored rectangles represent the conserved motifs defined by MEME on four representative alternative assemblies of the variable region. The positional frequency of each nucleotide in each motif generated using Weblogo is depicted below the figure. The 39-bp palindrome sequence is contained within motif 10. Motifs 5 (yellow) and 10 (light gray) depict the core of the conserved element across strains. [file 1471-2164-7-60-S2.pdf]

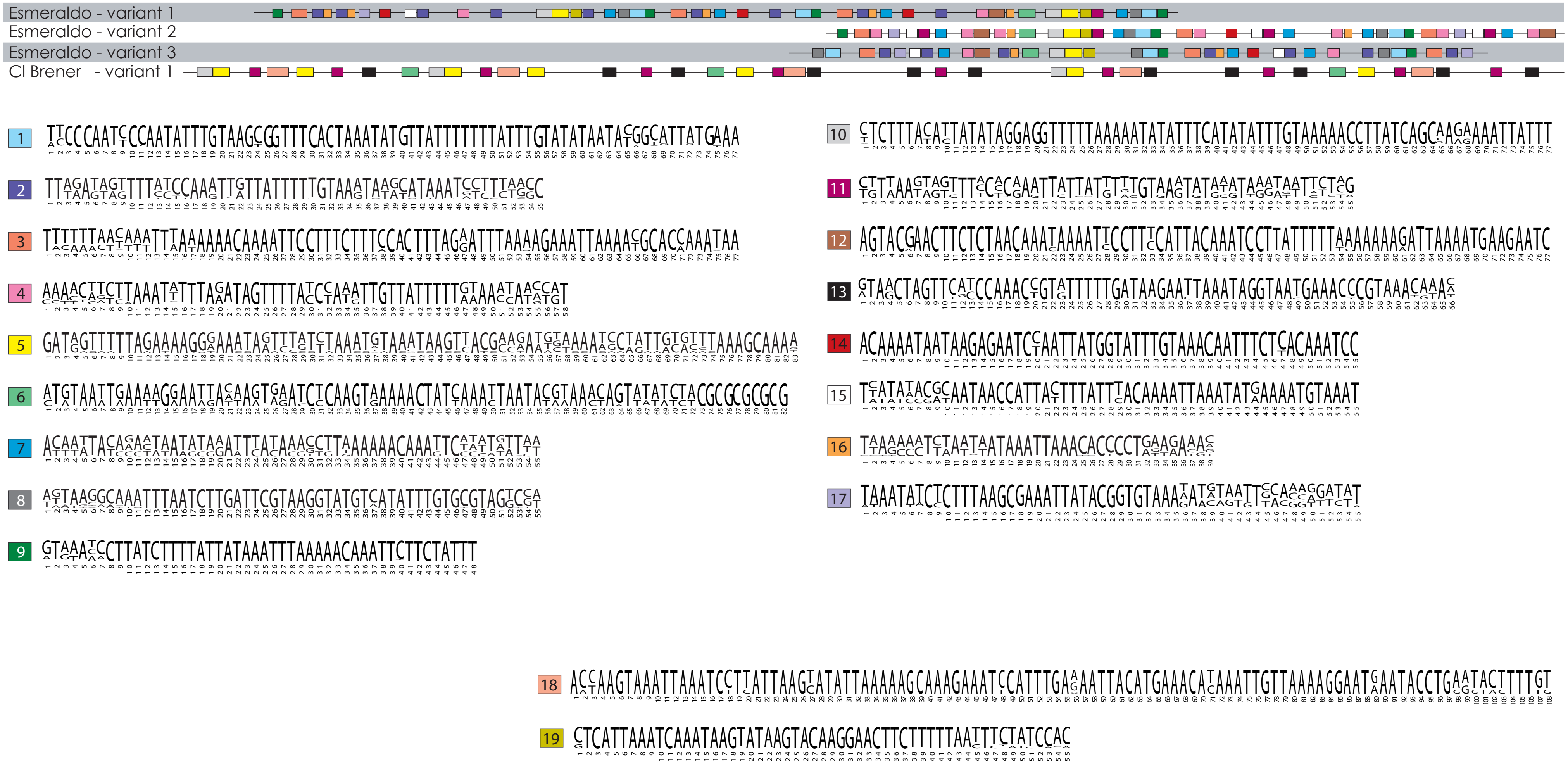

Esmeraldo - variant 1
Esmeraldo - variant 2
Esmeraldo - variant 3
Cl Brener - variant 1
1
2
3
4
5
6
7
8
9
10
11
12
13
14
15
16
17
18
19
