# Supplementary material for: Trypanosoma cruzi mitochondrial maxicircles display species- and strain-specific variation and a conserved element in the non-coding region
Source: BMC Genomics. 2006 Mar 22;7:60. doi: 10.1186/1471-2164-7-60 (PMC1559615; doi:10.1186/1471-2164-7-60)
Supplement: Additional File 3 — Conserved sequence elements of the maxicircle variable region. Alignment of conserved element sequence variants from CL Brener and Esmeraldo assemblies was generated by ClustalX. Consen indicates ClustalX consensus annotation. [file 1471-2164-7-60-S3.doc]

Conserved sequence elements of the maxicircle variable region

....|....| ....|....| ....|....| ....|....| ....|....| ....|....| ....|....| ....|....| ....|....|

10 20 30 40 50 60 70 80 90

**Esmo.7** TAATTGAAAA GGAATTATAA GTGAATCTCA AGTAAAAACT ATC-AAATTA ATACGTAAAC AGT-ATATCT ACGCGCGCGC GGATTAATA-

**Esmo.4** TAATTGAAAA GGAATTACAA GTRAATCTCA AGTAAAAACT ATC-AAATTA ATACGTAAAC AGT-ATATCT ACGCGCGCGC GGATTAATA-

**Esmo.2** TAATTGAAAA GGAATTATAA GTGAATCTCA AGTAAAAACT ATC-AAATTA ATACGTAAAC AGT-ATATCT ACGCGCGCGC GGATTAA---

**Esmo.6** TAATTGAAAA GGAATTATAA GTGAATCTCA AGTAAAAACT ATC-AAATTA ATACGTAAAC AGT-ATATCT ACGCGCGCGC GGATTAAGAT

**Esmo.5** TAATTGAAAA GGAATTACAA GTGAATCTCA AGTAAAAACT ATC-AAATTA ATACGTAAAC AAT-ATATCT ACGCGCGCGC GGATTAAT--

**Esmo.2** TAATTGAAAA GGAATTACAA GTGAATCTCA AGTAAAAACT ATC-AAATTA ATACGTAAAC AAT-ATATCT ACGCGCGCGC GGATTAAT--

**Esmo.8** TAATTGAAAA GGAATTACAA GTGAATCTCA A--------- ------ATTA ATACGTAAAC AAT-ATATCT ACGCGCGC-- GGATTAATT-

**Esmo.1** TAATTGAAAA GGAATTACAA GTGAATCTCA A--------- ------ATTA ATACGTAAAC AAT-ATATCT ACGCGCGC-- GGATTAATT-

**Esmo.3** TAATTGAAAA GGAATTACAA GTAAATCTCA AGTAAAAACT ATC-AAATTA ATACGTAAAC AGT-ATATCT ACGCGCGCGC GGATTAAT--

**CLB.2**  TAAATTGAAA AGAAT----A ATAGACCCCA AGTGAAAACT ATT-AAACTA ATATAAAACT AGTTATATCT -CGCGCGCGC GGATTAAAG-

**CLB.4**  TAAATTGAAA AGAAT----A ATAGATCCCA AGTGAAAACT ATTTAAACTA ATATAGAACT AGTTATATCT -CGCGCGCGC GGATTAAAG-

**CLB.3**  TAAATTGAAA AGAAT----A ACAGATCCCA AGTGAAAACT ATT-AAACTA ATATAAAACT AGTTATATCT -CGCGCGCGC GGATTAAA--

**CLB.5**  TAAATTGAAA AGAAT----A ACAGATCCCA AGTGAAAACT ATT-AAACTA ATATAAAACT AGTTGTATCT -CGCGTGCGC GGATTAAA--

**Consen** *** * *** **** * * * ** * * ** *** ** * * ***** **** ** *******

....|....| ....|....| ....|....| ....|....| ....|....| ....|....| ....|....| ....|....| ....|....|

100 110 120 130 140 150 160 170 180

**Esmo.7** --TATTATAA ACTAATAGAG TAAG-GGATC TGTGTTAATT CTGTTATTAC TCTTTACATT TTTACATTAT ATAGGAGGTT TTTAAAAATA

**Esmo.4** --T--TATAA ACTAATAGAG TAAG-GGATC TGTATTAATT CTGTTATTAC TCTTTACATT --------AT ATAGGAGGTT TTTAAAAATA

**Esmo.2** --TATTATAA ACTAATAGAG TAAG-GGATC TGTATTAATT CTGTTATTAC TCTTTACATT --------AT ATAGGAGGTT TTTAAAAATA

**Esmo.6** AATATTATAA ACTAATAGAG TAAG-GGATC TGTATTAATT CTGTTATTAC TCTTTACATT --------AT ATAGGAGGTT TTTAAAAATA

**Esmo.5** ---ATTATAA ACTAATAGAG TAAG-GGATC TGTATTAATT CTGTTATTAC TCTTTACATT --------AT ATAGGAGGTT TTTAAAAATA

**Esmo.2** ---ATTATAA ACTAATAGAG TAAG-GGATC TGTATTAATT CTGTTATTAC TCTTTACATT --------AT ATAGGAGGTT TTTAAAAATA

**Esmo.8** ---ATTATAA ACTAATAGAG TAAG-GGATC TGTATTAATT CTGTTATTAC TCTTTACATT --------AT ATAGGAGGTT TTTAAAAATA

**Esmo.1** ---ATTATAA ACTAATAGAG TAAG-GGATC TGTATTAATT CTGTTATTAC TCTTTACATT --------AT ATAGGAGGTT TTTAAAAATA

**Esmo.3** ---ATTATAA ACTAATAGAG TAAG-GGATC TGTATTAATT CTGTTATTAC TCTTTACATT --------AT ATAGGAGGTT TTTAAAAATA

**CLB.2**  ----TTATAA ATATATAGAG TAAGTGGATC TGTAATAGTT CTGTTATTAT TCTTTATACT --------AT ATAGGAGGTT TTTAAAAATA

**CLB.4**  ----TTATAA ATATATAGAG TAAGTGGATC TGTAATAGTT CTGTTATTAT TCTTTATACT --------AT ATAGGAGGTT TTTAAAAATA

**CLB.3**  ---------- ---------- ---------- ---------- ---------- ---------- ---------- ---------- -TTAAAAATA

**CLB.5**  ---------- ---------- ---------- ---------- ---------- ---------- ---------- ---------- ----GAAATA

**Consen** *****

....|....| ....|....| ....|....| ....|....| ....|....| ....|....| ....|....| ....|....| ....|....|

190 200 210 220 230 240 250 260 270

**Esmo.7** TATTTCATAT ATTTGTAAAA ACCTTAT-CA GCAAGAAAAT TATTTT-GAT GGTTTTTAGA AAAGGGAAAT AGTTTATCTA AATGTAAATA

**Esmo.4** TATTTCATAT ATTTGTAAAA ACCTTAT-CA GCGAGAAAAT TATTTT-GAT GGTTTTTAGA AAAGGGAAAT AGTTTATCTA AATRTAAATA

**Esmo.2** TATTTCATAT ATTTGTAAAA ACCTTAT-CA GCAAGAAAAT TATTTT-GAT GGTTTTTAGA AAAGGGAAAT AGTTTATCTA AATGTAAATA

**Esmo.6** TATTTCATAT ATTTGTAAAA ACCTTAT-CA GCMAGAAAAT TATTTT-GAT GGTTTTTAGA AAAGGGAAAT AGTTTATCTA AATRTAAATA

**Esmo.5** TATTTCATAT ATTTGTAAAA ACCTTAT-CA GCAAGAAAAT TATTTT-GAT AGTTTTTAGA AAAGGGAAAT AGTTCATCTA AATATAAATA

**Esmo.2** TATTTCATAT ATTTGTAAAA ACCTTAT-CA GCAAGAAAAT TATTTT-GAT AGTTTTTAGA AAAGGGAAAT AGTTCATCTA AATATAAATA

**Esmo.8** TATTTCATAT ATTTGTAAAA ACCTTAT-CA GCAAGAAAAT TATTTT-GAT GGTTTTTAGA AAAGGGAAAT AGTTTATCTA AATGTAAATA

**Esmo.1** TATTTCATAT ATTTGTAAAA ACCTTAT-CA GCAAGAAAAT TATTTT-GAT GGTTTTTAGA AAAGGGAAAT AGTTTATCTA AATGTAAATA

**Esmo.3** TATTTCATAT ATTTGTAAAA ACCTTAT-CA GCGAGAAAAT TATTCT-GAT GGTTTTTAGA AAAGGGAAAT AGTTTATCTA AATGTAAATA

**CLB.2**  TATTTCATAT ATTTGTAAAA ACCTTAT-CA GCAAAGAAAT TATTTTTGAT AGTTTTTAGA AAAGGAAAAT AATCTGTTTA AATGTAAATA

**CLB.4**  TATTTTATAT ATTTGTAAAA ACCTTAT-CA GCAAAGAAAT TATTTTTGAT AGTTTTTAGA AAAGGAAAAT AATCTGTTTA AATGTAAATA

**CLB.3**  TATTTCATAT ATTTGTAAAA ACCTTAT-CA GCAAAGAAAT TATTTTTGAT AGTTTTTAGA AAAGGAAAAT AATCTGTTTA AATGTAAGTA

**CLB.5**  TATWTCATAT ATTTGTAAAA ACCTTATACA GCAAAGAAAT TATTTTTGAT AGTTTTTAGA AAAGAAAAAT AATTCGTTTA AATGTAAATA

**Consen** *** * **** ********** ******* ** ** * **** **** * *** ********* **** **** * * * ** *** *** **

....|....| ....|....| ....|....| ....|....| ....|....| ....|....| ....|....| ...

280 290 300 310 320 330 340

**Esmo.7** AGTTACGA-A GATGCAAAAT CCTATTGTGT TTAAAGCAAA ATG------- ---TCATTAA ATCAAATAAG TAT

**Esmo.4** AGTTACGA-A GATGTAAAAT CCTATTGTGT CTAAAGCAAA ATGATATAAA TGCTCATTAA ATCAAATAAG TAT

**Esmo.2** AGTTACGA-A GATGCAAAAT CCTATTGTGT TTAAAGCAAA ATG------- ---TCATTAA ATCAAATAAG TAT

**Esmo.6** AGTTACGA-A GATGYAAAAT CCTATTGTGT TTAAAGCAAA ATGATATAAA TGCTCATTAA ATCAAATAAG YAT

**Esmo.5** AGTTACGA-A GATGTAAAAT CCTATTGTGT TTAAAGCAAA ATGATATAAA TGCTCATTAA ATCAAATAAG TAT

**Esmo.2** AGTTACGA-A GATGTAAAAT CCTATTGTGT TTAAAGCAAA ATGATATAAA TGCTCATTAA ATCAAATAAG TAT

**Esmo.8** AGTTACGA-A GATGTAAAAT CCTATTGTGT TTAAAGCAAA ATGATA-AAA TGCTCATTAA ATCAAATAAG TAT

**Esmo.1** AGTTACGA-A GATGTAAAAT CCTATTGTGT TTAAAGCAAA ATGATA-AAA TGCTCATTAA ATCAAATAAG TAT

**Esmo.3** AGTTACGA-A GATGTAAAAT CCTATTGTGT CTAAAGCAAA ATGATATAAA TGCTCATTAA ATCAAATAAG TAT

**CLB.2**  AGTCACGCCA AATGTAAAAG CA-GTTACAC TCAAAGCAAA GCARCAACAA G--TCCTTA- -TCAAATAAG TAT

**CLB.4**  AGTCACGCCA AATGTAAAAG CA-GTTACAC TTAAAGCAAA RCAACAACAA G--TCCTTA- -TCAAATAAG TAT

**CLB.3**  AGTCACGCCA AATGTAAAAG CA-GTTACAC TTAAAGCAAA ACAACAATAA G--TCCTTA- -TCAAATAAG TAT

**CLB.5**  AGTCACGCCA AATGTAAAAG CA-GTTACAC TTAAAGCAAA ACAACAATAA G--TCCTTA- -TCAAATAAG YAT

**Consen** *** *** * *** **** * ** ******** ** *** ********* **
