# Supplementary material for: Trypanosoma cruzi mitochondrial maxicircles display species- and strain-specific variation and a conserved element in the non-coding region
Source: BMC Genomics. 2006 Mar 22;7:60. doi: 10.1186/1471-2164-7-60 (PMC1559615; doi:10.1186/1471-2164-7-60)
Supplement: Additional File 5 — Strain-specific indels in non-edited genes resulting in frameshifts. ND5, MURF1 and MURF2 predicted protein alignments and partial coding region alignments displaying effects of indel mutations. Arrows indicate the position of indel mutations. Black highlighting indicates regions of frameshifts relative to T. brucei. Premature termination codons are underlined in DNA alignments and depicted as stars in protein sequences. MURF2 protein sequence is based on predicted 5' editing conserved with T. brucei with insertion of 24 U's up to nt 45. [file 1471-2164-7-60-S5.ppt]

## Slide 1
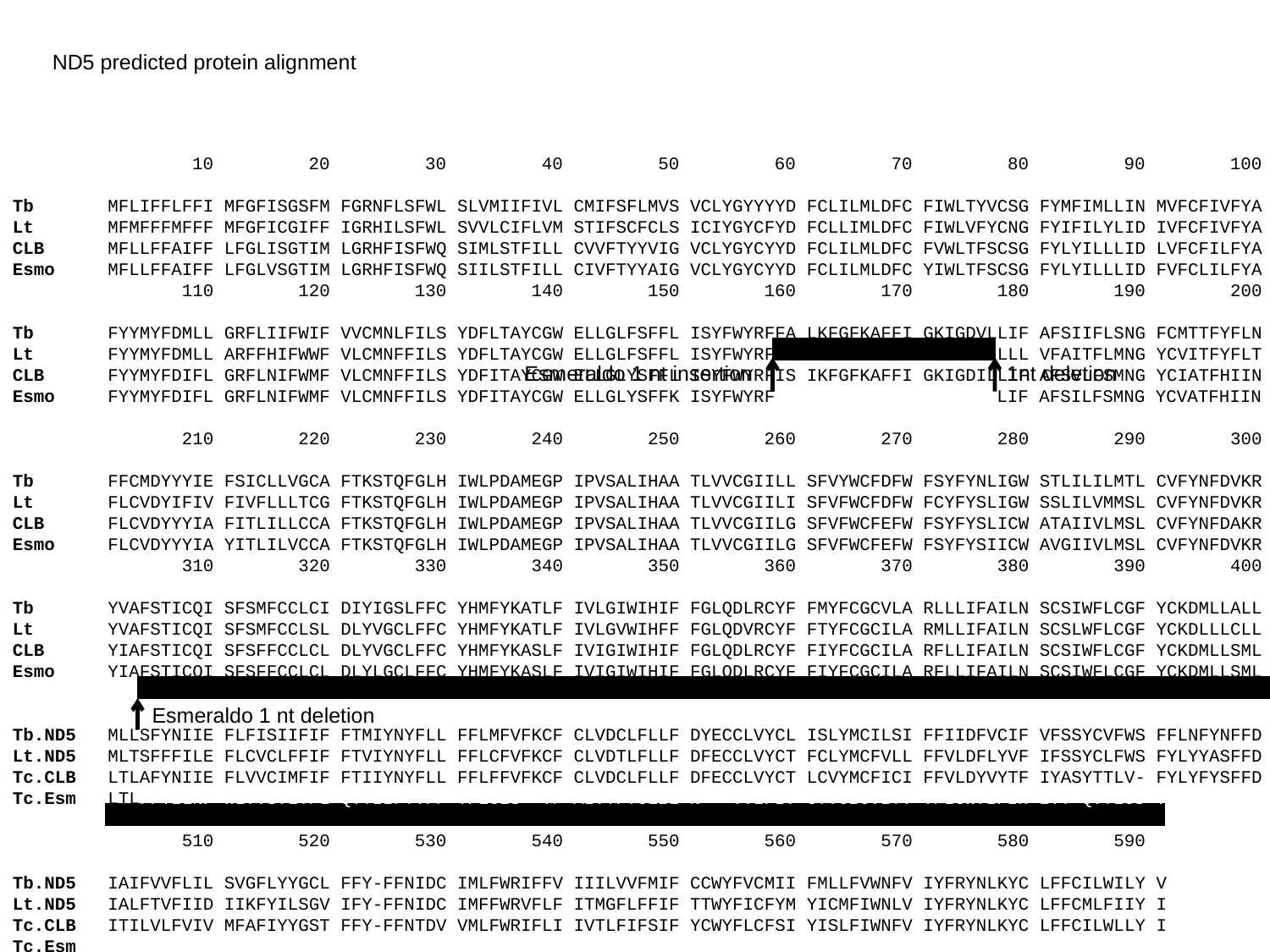

ND5 predicted protein alignment
 10 20 30 40 50 60 70 80 90 100
Tb MFLIFFLFFI MFGFISGSFM FGRNFLSFWL SLVMIIFIVL CMIFSFLMVS VCLYGYYYYD FCLILMLDFC FIWLTYVCSG FYMFIMLLIN MVFCFIVFYA
Lt MFMFFFMFFF MFGFICGIFF IGRHILSFWL SVVLCIFLVM STIFSCFCLS ICIYGYCFYD FCLLIMLDFC FIWLVFYCNG FYIFILYLID IVFCFIVFYA
CLB MFLLFFAIFF LFGLISGTIM LGRHFISFWQ SIMLSTFILL CVVFTYYVIG VCLYGYCYYD FCLILMLDFC FVWLTFSCSG FYLYILLLID LVFCFILFYA
Esmo MFLLFFAIFF LFGLVSGTIM LGRHFISFWQ SIILSTFILL CIVFTYYAIG VCLYGYCYYD FCLILMLDFC YIWLTFSCSG FYLYILLLID FVFCLILFYA
 110 120 130 140 150 160 170 180 190 200
Tb FYYMYFDMLL GRFLIIFWIF VVCMNLFILS YDFLTAYCGW ELLGLFSFFL ISYFWYRFFA LKFGFKAFFI GKIGDVLLIF AFSIIFLSNG FCMTTFYFLN
Lt FYYMYFDMLL ARFFHIFWWF VLCMNFFILS YDFLTAYCGW ELLGLFSFFL ISYFWYRFYA LKFGFKAFFI SKVGDVLLLL VFAITFLMNG YCVITFYFLT
CLB FYYMYFDIFL GRFLNIFWMF VLCMNFFILS YDFITAYCGW ELLGLYSFFL ISYFWYRFIS IKFGFKAFFI GKIGDILLIF AFSVLFSMNG YCIATFHIIN
Esmo FYYMYFDIFL GRFLNIFWMF VLCMNFFILS YDFITAYCGW ELLGLYSFFK ISYFWYRFFF YQIRF*SIFY R*NRRCILIF AFSILFSMNG YCVATFHIIN
 210 220 230 240 250 260 270 280 290 300
Tb FFCMDYYYIE FSICLLVGCA FTKSTQFGLH IWLPDAMEGP IPVSALIHAA TLVVCGIILL SFVYWCFDFW FSYFYNLIGW STLILILMTL CVFYNFDVKR
Lt FLCVDYIFIV FIVFLLLTCG FTKSTQFGLH IWLPDAMEGP IPVSALIHAA TLVVCGIILI SFVFWCFDFW FCYFYSLIGW SSLILVMMSL CVFYNFDVKR
CLB FLCVDYYYIA FITLILLCCA FTKSTQFGLH IWLPDAMEGP IPVSALIHAA TLVVCGIILG SFVFWCFEFW FSYFYSLICW ATAIIVLMSL CVFYNFDAKR
Esmo FLCVDYYYIA YITLILVCCA FTKSTQFGLH IWLPDAMEGP IPVSALIHAA TLVVCGIILG SFVFWCFEFW FSYFYSIICW AVGIIVLMSL CVFYNFDVKR
 310 320 330 340 350 360 370 380 390 400
Tb YVAFSTICQI SFSMFCCLCI DIYIGSLFFC YHMFYKATLF IVLGIWIHIF FGLQDLRCYF FMYFCGCVLA RLLLIFAILN SCSIWFLCGF YCKDMLLALL
Lt YVAFSTICQI SFSMFCCLSL DLYVGCLFFC YHMFYKATLF IVLGVWIHFF FGLQDVRCYF FTYFCGCILA RMLLIFAILN SCSLWFLCGF YCKDLLLCLL
CLB YIAFSTICQI SFSFFCCLCL DLYVGCLFFC YHMFYKASLF IVIGIWIHIF FGLQDLRCYF FIYFCGCILA RFLLIFAILN SCSIWFLCGF YCKDMLLSML
Esmo YIAFSTICQI SFSFFCCLCL DLYLGCLFFC YHMFYKASLF IVIGIWIHIF FGLQDLRCYF FIYFCGCILA RFLLIFAILN SCSIWFLCGF YCKDMLLSML
 410 420 430 440 450 460 470 480 490 500
Tb.ND5 MLLSFYNIIE FLFISIIFIF FTMIYNYFLL FFLMFVFKCF CLVDCLFLLF DYECCLVYCL ISLYMCILSI FFIIDFVCIF VFSSYCVFWS FFLNFYNFFD
Lt.ND5 MLTSFFFILE FLCVCLFFIF FTVIYNYFLL FFLCFVFKCF CLVDTLFLLF DFECCLVYCT FCLYMCFVLL FFVLDFLYVF IFSSYCLFWS FYLYYASFFD
Tc.CLB LTLAFYNIIE FLVVCIMFIF FTIIYNYFLL FFLFFVFKCF CLVDCLFLLF DFECCLVYCT LCVYMCFICI FFVLDYVYTF IYASYTTLV- FYLYFYSFFD
Tc.Esm LTLFFTILNF WLYVSYLYFL QYTIIFYYFF YFLCLS--VF AIVYFYCLIL N---VVLFIV CYVCICVLYV YFLCWTIFIH LYT-QVTLCS YFIYIFIVFL
 510 520 530 540 550 560 570 580 590
Tb.ND5 IAIFVVFLIL SVGFLYYGCL FFY-FFNIDC IMLFWRIFFV IIILVVFMIF CCWYFVCMII FMLLFVWNFV IYFRYNLKYC LFFCILWILY V
Lt.ND5 IALFTVFIID IIKFYILSGV IFY-FFNIDC IMFFWRVFLF ITMGFLFFIF TTWYFICFYM YICMFIWNLV IYFRYNLKYC LFFCMLFIIY I
Tc.CLB ITILVLFVIV MFAFIYYGST FFY-FFNTDV VMLFWRIFLI IVTLFIFSIF YCWYFLCFSI YISLFIWNFV IYFRYNLKYC LFFCILWLLY I
Tc.Esm ILLYWYCLCV HLCIMEVLFL FFYGCCYVSL KSIFNYNNTV YIFDILLLIF SMFFYICIFI YL-----KFC YIFQIFKILF VFLYVMIVVY
Esmeraldo 1 nt insertion 1nt deletion
Esmeraldo 1 nt deletion

## Slide 2
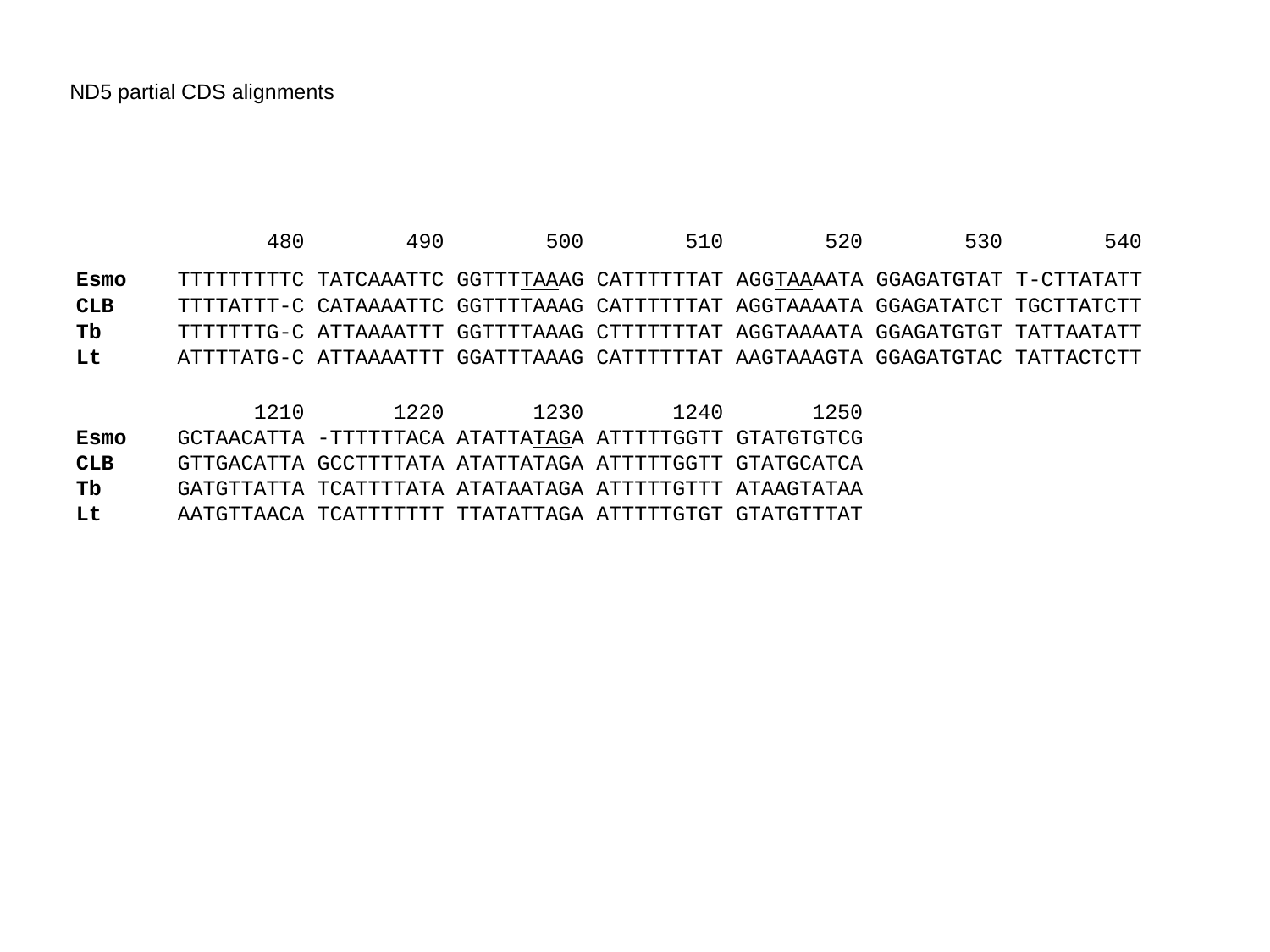

ND5 partial CDS alignments
 480 490 500 510 520 530 540
Esmo TTTTTTTTTC TATCAAATTC GGTTTTAAAG CATTTTTTAT AGGTAAAATA GGAGATGTAT T-CTTATATT
CLB TTTTATTT-C CATAAAATTC GGTTTTAAAG CATTTTTTAT AGGTAAAATA GGAGATATCT TGCTTATCTT
Tb TTTTTTTG-C ATTAAAATTT GGTTTTAAAG CTTTTTTTAT AGGTAAAATA GGAGATGTGT TATTAATATT
Lt ATTTTATG-C ATTAAAATTT GGATTTAAAG CATTTTTTAT AAGTAAAGTA GGAGATGTAC TATTACTCTT
 1210 1220 1230 1240 1250
Esmo GCTAACATTA -TTTTTTACA ATATTATAGA ATTTTTGGTT GTATGTGTCG
CLB GTTGACATTA GCCTTTTATA ATATTATAGA ATTTTTGGTT GTATGCATCA
Tb GATGTTATTA TCATTTTATA ATATAATAGA ATTTTTGTTT ATAAGTATAA
Lt AATGTTAACA TCATTTTTTT TTATATTAGA ATTTTTGTGT GTATGTTTAT

## Slide 3
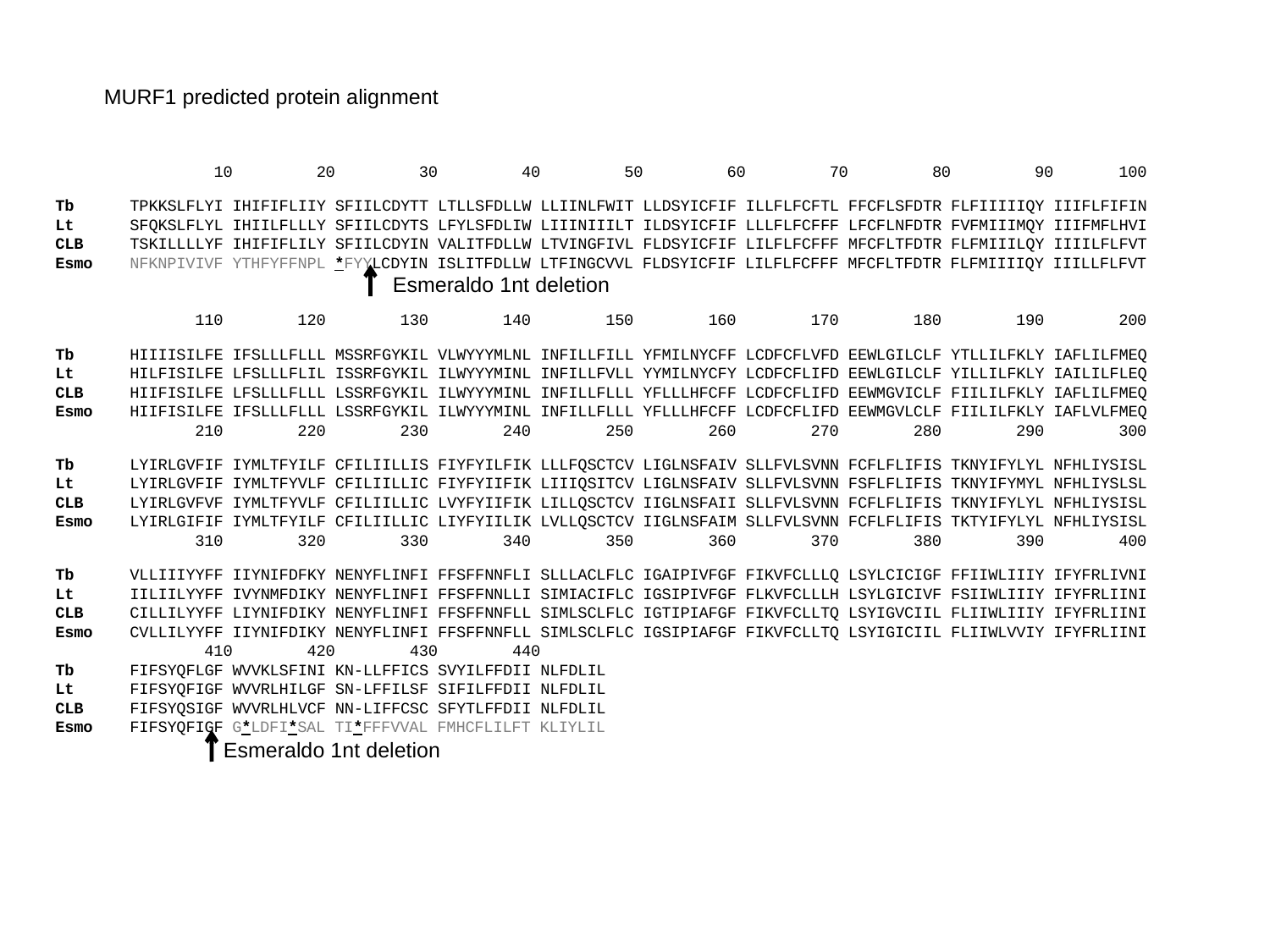

MURF1 predicted protein alignment
# 10 20 30 40 50 60 70 80 90 100
Tb TPKKSLFLYI IHIFIFLIIY SFIILCDYTT LTLLSFDLLW LLIINLFWIT LLDSYICFIF ILLFLFCFTL FFCFLSFDTR FLFIIIIIQY IIIFLFIFIN
Lt SFQKSLFLYL IHIILFLLLY SFIILCDYTS LFYLSFDLIW LIIINIIILT ILDSYICFIF LLLFLFCFFF LFCFLNFDTR FVFMIIIMQY IIIFMFLHVI
CLB TSKILLLLYF IHIFIFLILY SFIILCDYIN VALITFDLLW LTVINGFIVL FLDSYICFIF LILFLFCFFF MFCFLTFDTR FLFMIIILQY IIIILFLFVT
Esmo NFKNPIVIVF YTHFYFFNPL *FYYLCDYIN ISLITFDLLW LTFINGCVVL FLDSYICFIF LILFLFCFFF MFCFLTFDTR FLFMIIIIQY IIILLFLFVT
 110 120 130 140 150 160 170 180 190 200
Tb HIIIISILFE IFSLLLFLLL MSSRFGYKIL VLWYYYMLNL INFILLFILL YFMILNYCFF LCDFCFLVFD EEWLGILCLF YTLLILFKLY IAFLILFMEQ
Lt HILFISILFE LFSLLLFLIL ISSRFGYKIL ILWYYYMINL INFILLFVLL YYMILNYCFY LCDFCFLIFD EEWLGILCLF YILLILFKLY IAILILFLEQ
CLB HIIFISILFE LFSLLLFLLL LSSRFGYKIL ILWYYYMINL INFILLFLLL YFLLLHFCFF LCDFCFLIFD EEWMGVICLF FIILILFKLY IAFLILFMEQ
Esmo HIIFISILFE IFSLLLFLLL LSSRFGYKIL ILWYYYMINL INFILLFLLL YFLLLHFCFF LCDFCFLIFD EEWMGVLCLF FIILILFKLY IAFLVLFMEQ
 210 220 230 240 250 260 270 280 290 300
Tb LYIRLGVFIF IYMLTFYILF CFILIILLIS FIYFYILFIK LLLFQSCTCV LIGLNSFAIV SLLFVLSVNN FCFLFLIFIS TKNYIFYLYL NFHLIYSISL
Lt LYIRLGVFIF IYMLTFYVLF CFILIILLIC FIYFYIIFIK LIIIQSITCV LIGLNSFAIV SLLFVLSVNN FSFLFLIFIS TKNYIFYMYL NFHLIYSLSL
CLB LYIRLGVFVF IYMLTFYVLF CFILIILLIC LVYFYIIFIK LILLQSCTCV IIGLNSFAII SLLFVLSVNN FCFLFLIFIS TKNYIFYLYL NFHLIYSISL
Esmo LYIRLGIFIF IYMLTFYILF CFILIILLIC LIYFYIILIK LVLLQSCTCV IIGLNSFAIM SLLFVLSVNN FCFLFLIFIS TKTYIFYLYL NFHLIYSISL
 310 320 330 340 350 360 370 380 390 400
Tb VLLIIIYYFF IIYNIFDFKY NENYFLINFI FFSFFNNFLI SLLLACLFLC IGAIPIVFGF FIKVFCLLLQ LSYLCICIGF FFIIWLIIIY IFYFRLIVNI
Lt IILIILYYFF IVYNMFDIKY NENYFLINFI FFSFFNNLLI SIMIACIFLC IGSIPIVFGF FLKVFCLLLH LSYLGICIVF FSIIWLIIIY IFYFRLIINI
CLB CILLILYYFF LIYNIFDIKY NENYFLINFI FFSFFNNFLL SIMLSCLFLC IGTIPIAFGF FIKVFCLLTQ LSYIGVCIIL FLIIWLIIIY IFYFRLIINI
Esmo CVLLILYYFF IIYNIFDIKY NENYFLINFI FFSFFNNFLL SIMLSCLFLC IGSIPIAFGF FIKVFCLLTQ LSYIGICIIL FLIIWLVVIY IFYFRLIINI
 410 420 430 440
Tb FIFSYQFLGF WVVKLSFINI KN-LLFFICS SVYILFFDII NLFDLIL
Lt FIFSYQFIGF WVVRLHILGF SN-LFFILSF SIFILFFDII NLFDLIL
CLB FIFSYQSIGF WVVRLHLVCF NN-LIFFCSC SFYTLFFDII NLFDLIL
Esmo FIFSYQFIGF G*LDFI*SAL TI*FFFVVAL FMHCFLILFT KLIYLIL
Esmeraldo 1nt deletion
Esmeraldo 1nt deletion

## Slide 4
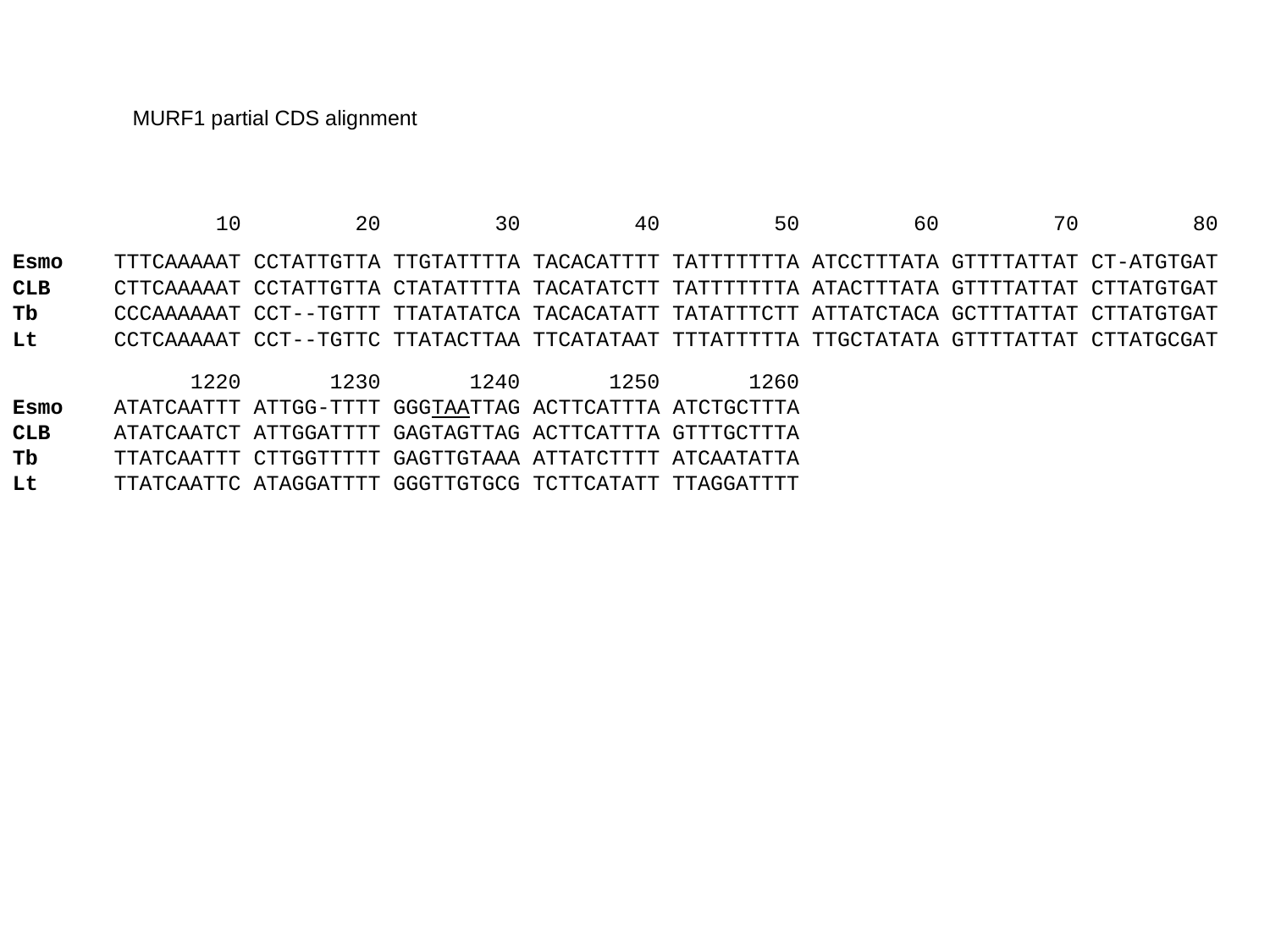

# MURF1 partial CDS alignment
 10 20 30 40 50 60 70 80
Esmo TTTCAAAAAT CCTATTGTTA TTGTATTTTA TACACATTTT TATTTTTTTA ATCCTTTATA GTTTTATTAT CT-ATGTGAT
CLB CTTCAAAAAT CCTATTGTTA CTATATTTTA TACATATCTT TATTTTTTTA ATACTTTATA GTTTTATTAT CTTATGTGAT
Tb CCCAAAAAAT CCT--TGTTT TTATATATCA TACACATATT TATATTTCTT ATTATCTACA GCTTTATTAT CTTATGTGAT
Lt CCTCAAAAAT CCT--TGTTC TTATACTTAA TTCATATAAT TTTATTTTTA TTGCTATATA GTTTTATTAT CTTATGCGAT
 1220 1230 1240 1250 1260
Esmo ATATCAATTT ATTGG-TTTT GGGTAATTAG ACTTCATTTA ATCTGCTTTA
CLB ATATCAATCT ATTGGATTTT GAGTAGTTAG ACTTCATTTA GTTTGCTTTA
Tb TTATCAATTT CTTGGTTTTT GAGTTGTAAA ATTATCTTTT ATCAATATTA
Lt TTATCAATTC ATAGGATTTT GGGTTGTGCG TCTTCATATT TTAGGATTTT

## Slide 5
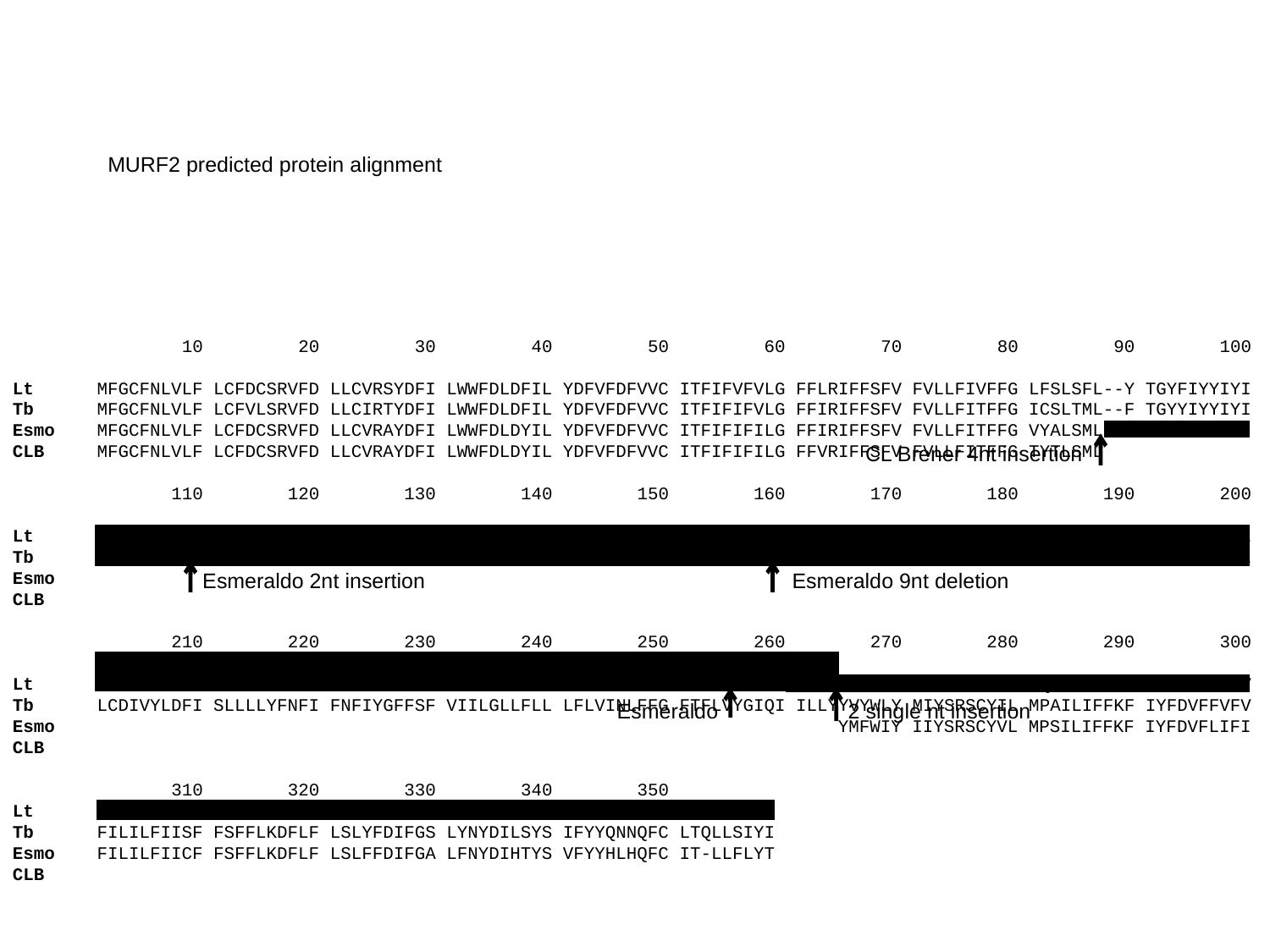

# MURF2 predicted protein alignment
 10 20 30 40 50 60 70 80 90 100
Lt MFGCFNLVLF LCFDCSRVFD LLCVRSYDFI LWWFDLDFIL YDFVFDFVVC ITFIFVFVLG FFLRIFFSFV FVLLFIVFFG LFSLSFL--Y TGYFIYYIYI
Tb MFGCFNLVLF LCFVLSRVFD LLCIRTYDFI LWWFDLDFIL YDFVFDFVVC ITFIFIFVLG FFIRIFFSFV FVLLFITFFG ICSLTML--F TGYYIYYIYI
Esmo MFGCFNLVLF LCFDCSRVFD LLCVRAYDFI LWWFDLDYIL YDFVFDFVVC ITFIFIFILG FFIRIFFSFV FVLLFITFFG VYALSML--Y TGYYIFYIYI
CLB MFGCFNLVLF LCFDCSRVFD LLCVRAYDFI LWWFDLDYIL YDFVFDFVVC ITFIFIFILG FFVRIFFSFV FVLLFITFFG TYTLSMLYIY RLLYIL*HIY
 110 120 130 140 150 160 170 180 190 200
Lt LYNFICYFFC F--SIALYYI EFFTYLLCFI FIDFISFSNH LISYFGIINM FNVIFCSYLF CLFYFIICFI FCFIFFVIRC LFVIIYDFLF FNFDIYISFL
Tb LYNFICFFFA FGINFLIYYI EFFIFITFHI FFDFISFSNY IYNYFGILYM FNVMFCAYLF CLFYFVIYFL FCFIFFVIRC LFIVIMDFLF FNFDIFVSIL
Esmo LYNFICFFFS H--LVSIFWY IIWNFLCLYY FIYFLTFVFH VLSIIFLVCY LHLILYFVRI CFVYFILFYF VLYFLFVVYL LYLIFY-FLI LIFSYHWRYA
CLB IIFHMFLFYV RYFSDILFGI FYVYIILSVF WFYKFFMFYL FFWSILHIYH ILYVFVLLIL FYNFFFILFY IFCNTLFICN CIR---LFIF FRYFYIINVM
 210 220 230 240 250 260 270 280 290 300
Lt MCDIIYIDYI CFLLIYFGFI FSFITGFFCF IFVLNYVFLV LFFVLALFFG FLFLSYGLFT FLIYYFFWLY IIYSRSCFIL LQSVVIFFKF LYFDVFFIFV
Tb LCDIVYLDFI SLLLLYFNFI FNFIYGFFSF VIILGLLFLL LFLVINLFFG FTFLVYGIQI ILLYYVYWLY MIYSRSCYIL MPAILIFFKF IYFDVFFVFV
Esmo TYSMI----- -LFVFYYISI ISQIS-YMAF IVLL-YDFFC YYIWYSIYIL DFFYNIWITI TLISYMFWIY IIYSRSCYVL MPSILIFFKF IYFDVFLIFI
CLB WCTVYR---- --FRVFLNIV FLYNKFHIWI LLFHNYIRVN FFIIIHGTFI FWFFFYNLWI NIFSDKLYIL NIYNIKLLYI NAVYFNIFVY LFWCFFNIYI
 310 320 330 340 350
Lt FLLILFIICF FGFFLKDFLF LNIFFDMFSV LLIYDVNNYS AFYNSYNQFC VTQLLAVYI
Tb FILILFIISF FSFFLKDFLF LSLYFDIFGS LYNYDILSYS IFYYQNNQFC LTQLLSIYI
Esmo FILILFIICF FSFFLKDFLF LSLFFDIFGA LFNYDIHTYS VFYYHLHQFC IT-LLFLYT
CLB YINFVHNMFF F-FFKRFFIF VIVFWYIWST VLRY-IHILC ILLPFTPILC NAIIISLYV
CL Brener 4nt insertion
Esmeraldo 2nt insertion
Esmeraldo 9nt deletion
Esmeraldo 2 single nt insertion
